# Mutant Huntingtin Affects Diabetes and Alzheimer’s Markers in Human and Cell Models of Huntington’s Disease

**DOI:** 10.3390/cells8090962

**Published:** 2019-08-23

**Authors:** Gepoliano Chaves, John Stanley, Nader Pourmand

**Affiliations:** Department of Biomolecular Engineering, University of California, Santa Cruz, CA 95064, USA

**Keywords:** sortilins, diabetes, cancer, neurodegeneration, Huntington’s Disease, GWAS, RNA-seq, ST14A cells, linkage, SORCS1, SORCS2, SORCS3, SORLA, SORT1, HLA, MHC

## Abstract

A higher incidence of diabetes was observed among family members of individuals affected by Huntington’s Disease with no follow-up studies investigating the genetic nature of the observation. Using a genome-wide association study (GWAS), RNA sequencing (RNA-Seq) analysis and western blotting of *Rattus norvegicus* and human, we were able to identify that the gene family of sortilin receptors was affected in Huntington’s Disease patients. We observed that less than 5% of SNPs were of statistical significance and that sortilins and HLA/MHC gene expression or SNPs were associated with mutant huntingtin (mHTT). These results suggest that ST14A cells derived from *R. norvegicus* are a reliable model of HD, since sortilins were identified through analysis of the transcriptome in these cells. These findings help highlight the genes involved in mechanisms targeted by diabetes drugs, such as glucose transporters as well as proteins controlling insulin release related to mHTT. To the best of our knowledge, this is the first GWAS using RNA-Seq data from both ST14A rat HD cell model and human Huntington’s Disease.

## 1. Introduction

### 1.1. Biology of Huntingtin and Identification of DNA Polymorphism Causing HD

Huntington’s disease (HD), a neurodegeneration characterized by motor, cognitive and psychiatric symptoms is caused by an unstable expansion (CAG) in a polyglutamine (polyQ) tract in the N-terminal of the huntingtin (HTT) gene. This condition is triggered when the number of CAG repeats on exon 1 of *HTT* exceeds 39, while 36-39 CAGs repeats result in an uncertain incidence of the disease [1]. Features of the genetic inheritance of CAG repeats in *HTT* were unknown for more than 200 years; George Huntington described Huntington’s chorea in 1872 and linkage mapping of the genomic region to HD occurred in 1993 [1,2]. HTT is highly conserved from flies to mammals and the N-terminal is the most extensively studied region of the protein due to the CAG expansion on exon 1. HTT also contains HEAT repeats which play an important role in protein-protein interactions. HEAT repeats are a common helical motif in the Huntingtin protein, Elongation Factor 3, protein phosphatase 2A, and TOR1 [3].

Tools such as the restriction fragment length polymorphism (RFLP) have been extensively used in determining DNA polymorphisms of HTT inherited in HD families [4], but rarely used to study other parts of the genome of HD individuals. RFLP was used in linkage disequilibrium studies that characterized the genetic pattern of CAG repeats in *HTT* in HD families. The reason for the lack of studies on the overall genome of HD individuals after characterization of mutant HTT is the assumption that no other genomic region influences human HD more than *HTT*. A study of SNPs in different parts of the genome of HD individuals may help explain different genetic phenomena including increased prevalence of diabetes among families affected with HD and cases where the CAG abnormal expansion was not present in the father nor in the mother, but manifested in the descendants [5,6,7]. Due to the great number of HTT molecular partners in events such as DNA and RNA metabolism, endocytosis, subcellular trafficking and cellular homeostasis, genetic diversity of several proteins that interact with HTT may affect prevalence these conditions among HD patients and their families.

Saudou and Humbert reviewed the interaction of HTT with proteins of the molecular motor machinery such as dynein, dynactin and kinesin and the role of HEAT repeats allowing HTT to serve as a scaffolding protein in these biological processes [8]. Zala et al., from the same group, found that the HTT-dependent transport of GADPH, a gene from the glycolysis pathway, provides energy to sustain the fast transport of vesicles using the cytoskeleton [9]. These studies support the notion that HTT acts as a protein important in the transport of vesicles, also influencing cellular endocytosis and exocytosis.

### 1.2. Neurodegeneration and Metabolic Diseases

Endocytosis and exocytosis are cellular mechanisms that maintain the homeostasis of a cell and play a direct role in neurodegenerations and metabolic diseases. Endocytosis and exocytosis influence Type 1 Diabetes (T1D) and Type 2 Diabetes (T2D) in different ways. T1D or insulin-dependent diabetes, features autoantibodies attacking proteins in the membrane of pancreatic cells. Problems in the processing of antigens transported intracellularly by components such as phagosome, proteasome, Golgi apparatus, ER, endosomes and the cell membrane are often associated with T1D. It has been observed that glutamate decarboxylase, zinc transporter-8, IA-2, tetraspanin 7 and insulin act as humoral autoantigens in T1D [10]. T2D, on the other hand, is characterized by a lack of response to insulin in cells that need to use glucose, such as muscle cells, liver cells and adipocytes, even with the production of the hormone in the pancreas. In cells of patients with T2D, glucose transporters and proteins involved in the translocation of glucose transporters do not correctly respond to the insulin signal. This results in a poor cellular uptake of glucose from the blood stream.

Among components involved in the translocation of glucose receptors to the cell membrane, sortilins control sorting of proteins across different cellular compartments, including traffic between the Golgi apparatus and the cellular membrane [11]. Translocation of insulin to the secretory pathway following the release of the hormone in the blood stream is a cellular role performed with assistance of sortilins [12]. The first report of mHTT affecting diabetes phenotypes was made in 1972, when a higher prevalence of diabetes was detected among HD patients [6]. This observation was later supported by findings indicating increased chances of diabetes among family members of individuals affected with HD [7]. Since HTT is a scaffolding protein that connects cytoskeleton proteins to proteins of the endocytosis and exocytosis pathways, it is possible that mutant mHTT affects diabetes phenotypes related to these pathways. 

### 1.3. VPS10P-Domain Receptors or Sortilins: Regulators of Subcellular Protein Trafficking and Markers of Diabetes and Neurodegeneration

The vacuolar protein sorting 10 protein (VPS10P) domain is a 700-amino-acid motif first identified in Saccharomyces cerevisiae that directs the trafficking of lysosomal enzymes from the Golgi apparatus to the vacuole [13]. VPS10P-domain receptors in mammals contain five members; SORT1 (sortilin), SORLA, SORCS1, SORCS2 and SORCS3. These receptors bind and neutralize a variety of ligands such as trophic factors, neuropeptides, glucose receptor 4 (GLUT4) and the amyloid beta peptide between the trans-Golgi network, the endosomes and the plasma-membrane [11,14,15,16]. In macrophages, SORT1 binds other proteins from the Golgi apparatus causing the migration of vesicles using the retromer complex. Vesicles containing sortilins end up fusing to the phagosome of macrophages and are known to deliver specific proteins required for immunological control of *Mycobacterium tuberculosis* by macrophages [17]. Due to its involvement with subcellular transport and signaling transduction, Reuter referred to SORT1 as a dual function protein. As a transduction protein, SORT1 controls the translocation of epidermal growth factor ligands. As an intracellular protein transport controller, SORT1 acts in antigen presentation in dendritic cells, illustrating the role of sortilin genes with the immune system [18].

Considering the involvement of sortilin genes in immunity mediated by macrophages, sorting of cellular proteins, as well as our previous study indicating the up-regulation of sortilin SORCS1 in a cell model expressing mHTT [11,12,17,18,19], here focused on investigating the association of sortilins with Huntington’s Disease using human and rat RNA-Seq datasets. We explore the involvement of sortilins in an antigen presentation in HD and specifically provide evidence that proves sortilins are part of the genetic component leading to diabetes in human HD. 

## 2. Materials and Methods

### 2.1. Library Preparation and Sequencing

ST14A total RNA was extracted and converted to cDNA using the Smart-seq2 protocol [20]. cDNA was then processed with the Nextera XT DNA library preparation kit according to the manufacturer’s protocol (Illumina, San Diego, CA, USA). Libraries were purified and size-selected using the Nvigen Size Selection Kit according to the manufacturer’s protocol [21]. The bioanalyzer 2100 high-sensitivity DNA assay (Agilent, Santa Clara, CA, USA) was used to check on the size range. The functional library concentration was determined with the KAPA Biosystems library quantification kit. The libraries were denatured after quantification and loaded on Illumina HiSeq 2000 for sequencing.

### 2.2. Human and Rat GENOMES

We used the human genome assembly version GRCh37.p13 (hg19) available at the Ensembl Genome Browser to align the human sequencing files analyzed in this study [22]. The *Rattus norvegicus* reference genome version R_nor5.0 was used to map rat sequencing files [22]. Gene coordinates for isolation of genetic variants using Unix commands and dbSNP IDs were also acquired using the Ensembl Genome Browser website, when indicated.

### 2.3. Genome-Wide Association Study (GWAS)

The genome-wide association of RNA-Seq reads with Huntington’s disease was performed using the costumer Unix/bash script disclosed by Mohammed Khalfan of the New York University Genetics Core Facility [23]. Briefly, reads were aligned to the reference genome using BWA, sorted, converted to a .BAM file, duplicate marked and indexed using Picard. The genome analyses toolkit (GATK) developed and provided by the Broad Institute of Harvard and MIT was then used for realignment, variant calling, single nucleotide polymorphism (SNP), indel filtering and extraction, base quality score recalibration, and covariate analyses [24,25,26]. Knowing that GWAS strategies are relatively new in science here we use the notion of GWAS as revised by Visscher: “GWAS is an experimental design used to detect associations between genetic variants and traits in samples from populations”, to determine that the algorithm used by this report represents the first GWAS using RNA-Seq data from the HD cell model and human Huntington’s Disease [27]. Outputs of the GATK/GWAS pipeline were files including the variant call format (.vcf), containing the SNPs identified for processing described and reported in this document. A list of Unix commands used for analysis shown in this study are provided in the Supplementary Materials.

### 2.4. Gene Expression Omnibus (GEO) RNA-Seq Datasets

We used GEO datasets previously published by Labadorf et al. (2015) (GEO accession number GSE64810) [28], Lin et al. (2016) (GEO accession number GSM2100621) [29] and HD iPSC Consortium (2017) (GEO accession number GSE95344) [30]. In this manuscript, we refer to these datasets as Labadorf (2015), Lin (2016) and HD iPSC Consortium (2017) datasets respectively (publication year may or may not be indicated). Files .vcf and .count generated respectively by the GATK and DESeq2 pipelines are included in the Supplementary Materials (these files contain respectively the DNA genetic variants and the read counts detected by these pipelines).

### 2.5. Post-GATK/GWAS Processing

Several processing steps were required after completion of the GATK/GWAS pipeline. These steps were necessary to allow formatting of the output files for compliance with downstream software requirements and were accomplished using Unix commands depicted in the Supplementary Materials (this section includes a brief description of each Unix command used as well as extra results supporting findings described in the main document). Other than the Unix commands depicted in the Supplementary Materials, bcftools (version 1.9) [31], vcftools (version 0.1.14) [32] and (version 0.1.14) Plink [33] were used to adjust the format of files used in the analysis. Plink was used for the extraction of .ped and .map files.

### 2.6. Statistical Analysis

PLINK was used for statistical testing of the comparison cases versus controls. Fisher’s exact test was calculated as depicted in the command shown in the Supplementary Materials to compare the abundance of alleles in the cases and control individuals [33]. A *p*-value threshold of 0.05 was used to consider a genetic variant statistically significant in our GATK/GWAS pipeline.

### 2.7. Manhattan Plots

R package Qqman was used to plot *p*-values below 0.05, indicating the association between HD and the specific genetic variant detected in each genomic position, according to package instructions [34]. We highlighted SNPs located outside of the gene body up to 1,000,000 base-pairs upstream and downstream of the gene body. We considered that within this region there existed regulatory elements influencing the genetic products encoded by the gene body.

### 2.8. Linkage Visualization Using Haploview

PLINK was used to extract pedigree and .map files from .vcf files and to input Haploview using default parameters, as determined by the Broad Institute guidelines in the website [23] and original paper recommendations [35]. The haplotype block was determined using the Four Gametes Rule option available on Haploview. Linkage plots were constructed with the standard LD color scheme (D′/LOD). Color represents linkage, in the following order: red (LOD ≥ 2 and D′ = 1), pink (LOD ≥ 2 and D′ < 1), blue (LOD < 2 and D′ = 1), and white (LOD < 2 and D′ < 1).

### 2.9. Validation of GATK/GWAS DNA Variants Identification by Pyrosequencing

The NGS Pyrosequencing method was used as a genotyping validation of the GATK/GWAS algorithm used in this study as previously described [36,37]. 

## 3. Results

### 3.1. mHTT Is Associated with SORCS1 Protein Up-Regulation and Sortilins SNPs

To investigate whether genetic variants of sortilin genes were associated with human HD, we performed a GWAS in rat and human datasets using the Genome Analysis Tool Kit (GATK). Table 1 represents an overview of the significant SNPs detected in the entire RNA-Seq dataset as well as in the sortilins gene body and vicinity.

Based on the total number of SNPs identified, we were able to distinguish two groups of datasets; those with low genetic variance and datasets with high genetic variance. It was observed that 220,000 Single Nucleotide Polymorphisms were significantly associated with mHTT in the ST14A and HD iPSC Consortium data, whereas more than 2.6 million SNPs were significantly associated with mHTT in the Labadorf and Lin datasets (Table 1). 1.4–3.8% of all SNPs detected were significant in each dataset and this similarity indicates that variants were consistent across all groups that were analyzed.

**Figure 1:**
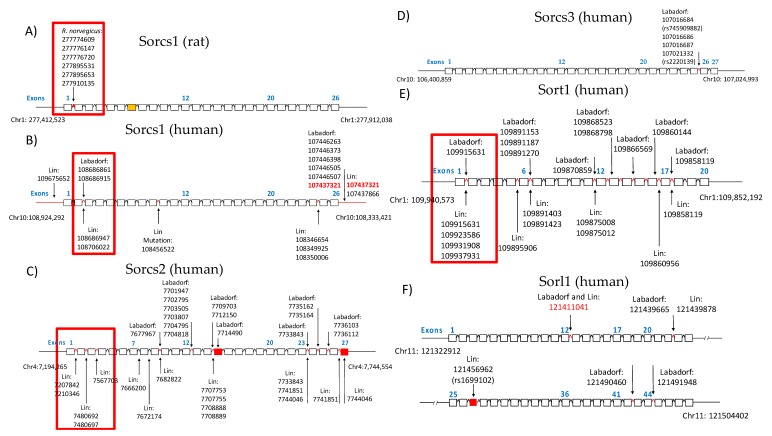
Organization of sortilins genomic regions showing SNPs detected by the GWAS/GATK pipeline across introns and exons. The dataset of the identification is indicated. (**A**) *SORCS1* gene organization in *R. norvegicus*. (**B**) *SORCS1* gene organization in human. (**C**) *SORCS2* gene organization in human. (**D**) *SORCS3* gene organization in human. (**E**) *SORT1* gene organization in human. (**F**) *SORL1* gene organization in human. Mutations inside the red squares represent SNPs in introns between exons that encode the VPS10P domain (Reitz 2013). Exons were indicated with their numbers in blue for identification.

We hypothesized that SNPs near the genes in the study could influence gene expression levels. To substantiate our hypothesis, we counted the number of SNPs in the gene body and regulatory regions. For this analysis, the gene regulatory region was defined as a body of ±1,000,000 bp nucleotides away from the gene body. We used a custom bash script to isolate the SNP positions (Supplementary Material, Command 1A). In the ST14A rat dataset, only variants of the SORCS1 gene were identified across the five possible sortilins (six SNPs in the gene body, 13 SNPs in the regulatory region) (Table 1). For a better visualization of sortilins SNPs observed in the GATK/GWAS pipeline, a plot was developed using the positions of the mutations relative to the sortilin gene organization and the dataset in which SNPs were identified as shown in Figure 1.

From these results, it can be observed that sortilins had mutations significantly associated with Huntington’s Disease (*p*-value < 0.05, Fisher’s exact test comparison) in the Labadorf dataset. Only SORCS3 and Sortilin-related (SORL1) did not show mutations flanking introns two and three, which are located between the exons encoding the VPS10P domain (Figure 1). For a list of all positions of significant SNPs associated with HD in this study, see the Supplementary Materials PLINK output.

**Figure 2:**
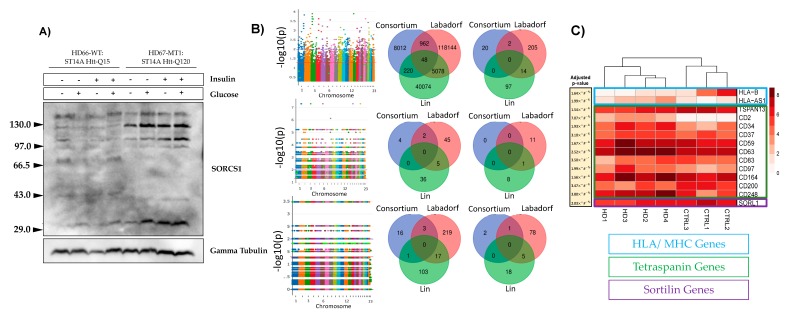
Influence of the mutant huntingtin (mHTT) on the expression and genetic variance of sortilin genes in *Rattus norvegicus* (A) and human (B). (**A**) Western blot of sortilin related VPS10 domain containing receptor 1 (SORCS1) protein in ST14A cells after overnight exposure to a growth medium containing glucose and bovine insulin; (**B**) Manhattan plots showing SNPs detected in Labdorf, Lin and HD iPSC Consortium datasets (top to bottom) (iPSC: induced Pluripotent Stem Cells). Venn diagrams depict SNPs detected in three human datasets analyzed per sortilin gene (HD iPSC Consortium, Blue; Labadorf 2015, Red; Lin 2016, Green). First the Venn diagram in the top shows SNPs in the three human datasets associated with HD (*p* < 0.05). The other five Venn diagrams show SNPs flanking the five sortilins (SORT1, SORL1, SORCS1, SORCS2 and SORCS3, from top to bottom); (**C**) RNA-Seq analysis using the DESeq2 R package on the HD iPSC Consortium dataset. Blue: MHC/HLA; Green: Tetraspanins; Purple: VPS10P (sortilins).

Figure 2A represents the agarose gel electrophoresis results. From the gel it is evident that the *SORCS1* protein levels were up-regulated in the mHTT-expressing cells, a phenomena that was reported earlier by our group [19]. To further access the effect of mHTT on the biology of sortilins, we investigated SNPs (Figure 2B) and the gene expression (Figure 2C) of sortilins associated with HD.

Labadorf et al. identified the expression of sortilins affected in HD individuals [28]. We believe that the HD iPSC Consortium dataset did not share common SNPs with the Labadorf and Lin datasets because the HD iPSC Consortium dataset was composed of “HD patient-derived iPSC lines with juvenile-onset CAG repeat expansions (60 and 109 repeats)” [30], a more genetically homogeneous group than the Labadorf and Lin datasets (Figure 2B). Further confirming our observations and those of the Labadorf group gene expression of sortilins are influenced by mHTT in the HD iPSC Consortium dataset (Figure 2C). Supplementary Table S1 shows the description of SNPs that were found significant across all human datasets (*p*-value < 0.05, Fisher’s exact test). Among the biological processes reported on the Supplementary Table S1, tetraspanin variants (illustrated in bold on Supplementary Table S1) indicate the association of mHTT with the four-transmembrane proteins. In the ST14A rat cells that we studied previously and here, the top up-regulated gene was *TSPAN8*, a transmembrane protein responsible for the organization of the HLA/MHC complex. This observation indicates the agreement between the findings concerning tetraspanins in the human and rat datasets analyzed in this study. 

**Figure 3:**
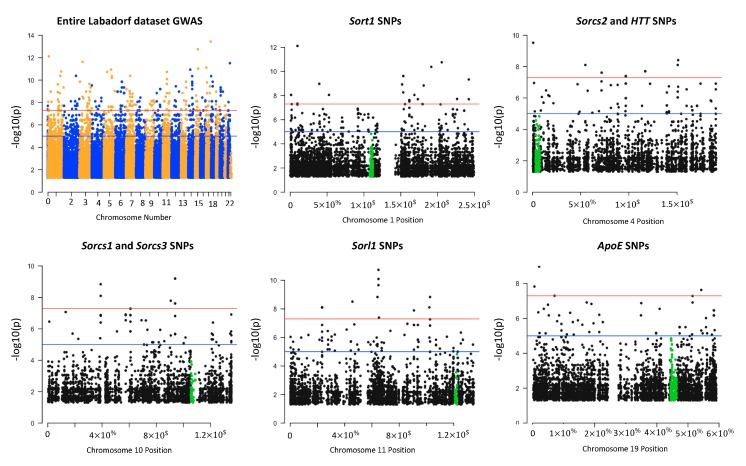
Manhattan plot visualization of SNPs found significant (*p*-value < 0.05) in regions near *HTT*, sortilins and *APOE* in the Labadorf 2015 dataset. Some of the genes were located in the same chromosome (*HTT* and *SORCS2* on chromosome 4, *SORCS1* and *SORCS3* on chromosome 10). When the chromosome was identical, significant SNPs were counted as SNPs located 1,000,000 bp upstream and downstream the gene border coordinates. The level of significance (negative log of *p*-value, y axis) of SNPs identified near the sortilin genes suggested that the genetic variation in sortilins have a significant impact on the Huntington’s disease (HD) pathology in this dataset.

To compare the distribution of SNPs identified near the sortilin regions in the GATK/GWAS pipeline exploited in this study with the overall SNPs flanking other genes, Manhattan plots were constructed with the significant (*p*-value < 0.05) 84,349 SNPs (Table 1) detected in the Labadorf dataset as shown in Figure 3. We included visualization of the *APOE* gene variants in Figure 3 due to its importance in neurodegenerations, Alzheimer’s Disease in particular. *APOE* genetic variants also affect human longevity due to the transport of lipids and risks associated with cardiovascular diseases [38].

Total SNPs detected between *HTT* and *SORCS2* on chromosome 4 (11746 SNPs, Supplementary Materials Table S6) were approximately 5X more SNPs than the region between *SORCS1* and *SORCS3* genes on chromosome 10 (1976 SNPs, Supplementary Materials Table S6). This is in agreement with the notion of the biological importance of the *HTT* locus as the Mendelian causative mutation of the HD pathology. Compared to the *APOE* locus which is highly polymorphic due to its activity in lipid metabolism, *HTT* carried at least 5X more SNP mutations than *APOE* (APOE carried 1863 total SNPs in its vicinity) (Figure 3, Supplementary Materials Table S6). We detected 665 SNPs significantly associated with HD in the vicinity of the HTT and sortilins genes (Supplementary Materials Table S6). Positions, alleles, allele frequencies and *p*-values of 665 SNPs between the HTT and sortilins genes significantly associated with HD are indicated in Supplementary Materials Table S7.

### 3.2. mHTT Affects Pathways Important for Immunological Function

By interrogating datasets analyzed for transcriptomic regulation and presence of SNPs associated with mHTT in specific genomic regions known to be involved with Type 1 and Type 2 Diabetes, we found that genes from the MHC/HLA locus, regarded as T1D markers [39], were down-regulated in human cells expressing mHTT in the HD iPSC Consortium dataset (HLA-B) (Figure 2C). MHC/HLA genes are responsible for the presentation of processed antigens to T-cells, and the activation of adaptive immune response, as the gene expression is turned on after a virus or bacterial infection or tumor mutation of proteins [40]. Figure 2C also shows that sortilin SORL1 and several tetraspanins are modulated by HD in the HD iPSC Consortium dataset. *SORL1* gene expression was down-regulated to approximately a third of the control values, and reached statistical significance (Supplementary Table S3). Other sortilins showed a similar trend, but not significance in the RNA-Seq analysis (Supplementary Table S3). The HLA-B gene showed a decrease in the gene expression average of more than 50X in the HD iPSC Consortium dataset (Supplementary Table S3).

The trend in the expression of the *SORL1* gene in human was opposite to the pattern of the protein expression of the *SORCS1* gene observed in ST14 rat cells, in that the *SORCS1* protein was up-regulated by almost 2X (for the quantification of protein bands shown on Figure 2A, see Supplementary Materials Figure S2 and Supplementary Table S5) in ST14A cells. These observations are based on the R package DESeq2 analysis [41] that we performed on the datasets, which was different than that performed by Labadorf et al. The *SORCS1* gene in ST14A cells was also up-regulated in the DESeq2 RNA-Seq analysis (Supplementary Table S4). In human datasets, sortilins showed a trend to be down-regulated in the HD iPSC Consortium dataset (Figure 2C, Supplementary Table S3), in the Labadorf dataset (Supplementary Table S6) and in the Lin 2016 dataset (Supplementary Table S7). However, the DESeq2 analysis was only detected as statistically significant, the difference in the expression levels of the *SORL1* gene in the HD iPSC Consortium dataset (Figure 2C), suggesting a gene expression influence of mHTT on *SORL1* similar to the trend detected previously in the AD for this receptor of Apolipoprotein E [42,43]. These findings suggest a need in characterization of gene expression alterations related to the immune function of sortilins in HD.

### 3.3. Validation of GATK/GWAS DNA Variants Identification by Pyrosequencing Methodology

**Figure 4:**
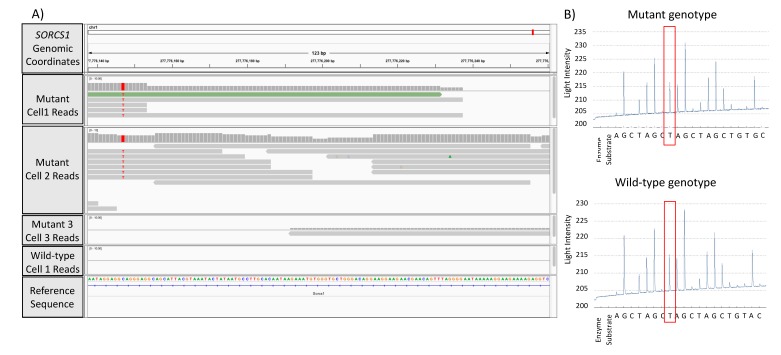
Visualization of mutations detected in the GATK/GWAS pipeline and validation of the pipeline by genomic DNA sequencing of samples using pyrosequencing. (**A**) Genome browser visualization of single-cell RNA-Seq reads spanning the *SORCS1* gene in mutant (mHTT) and wild-type cells. The figure shows mutant samples with zero, five and six reads spanning the *SORCS1* locus; (**B**) confirmation of detection of C>T substitution in the DNA sequence by pyrosequencing in *SORCS1*.

To validate the GATK/GWAS pipeline and aiming to establish the presence of an SNP as the result of RNA or DNA mutation, we performed a pyrosequencing detection of *SORCS1* SNPs in DNA extracted from ST14A cells carrying HTT and mHTT. To assess whether reads spanning SNPs loci in mutant cells were the result of cell population heterogeneity or clonal expansion of the SNP, we analyzed single cell data previously acquired in our lab from the same ST14A cells. Figure 4A shows the Integrative Genome Viewer (IGV) visualization of reads spanning the loci of the detected SNP. Figure 4B shows pyrosequencing results with mutation identified in genomic DNA.

Since the T allele was present in both the IGV visualization and DNA samples interrogated by pyrosequencing, we conclude that the variant was a DNA variant rather than a *SORCS1* mRNA variant. This result confirmed that this SNP mutation in *SORCS1* was identical to the mutation detected by the GATK/GWAS pipeline using ST14A mRNA molecules, a C > T DNA substitution (Figure 4B). 

### 3.4. Sortilin and HTT SNPs Identified by GATK/GWAS Pipeline of RNA-Seq Datasets of Human Origin Are in Linkage Disequilibrium

**Figure 5:**
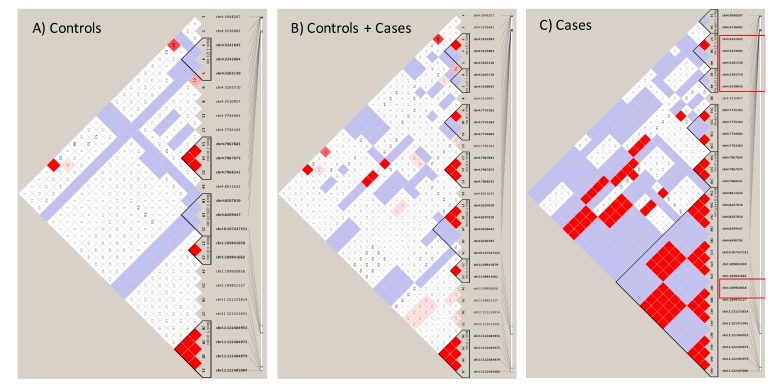
Linkage Disequilibrium of sortilin variants with *HTT* in human cases of the Labadorf dataset. Coordinates in chromosome 4 represent SNPs in the *HTT* and *SORCS2*. Coordinates in chromosome 10 represent SNPs in *SORCS1* and *SORCS3*. Coordinates in chromosome 1 and 11 represent SNPs in *SORT1* and *SORL1,* respectively. (**A**) Genotypes of control individuals; (**B**) genotypes of the control and cases combined; (**C**) genotypes of cases. High Linkage indicated by the shades of red between the *HTT* (position 3–3.2M of chromosome 4) and all sortilins.

We interrogated 166 SNPs reported in other studies about their presence in the Labadorf dataset. The Supplementary Table S2 shows SNPs identified in this GWAS study. 48 SNPs were present in the Labadorf dataset, indicating a detection rate close to 30% of well-documented publicly available sortilins SNPs in our study. Not necessarily all 48 SNPs had a significant *p*-value suggestive of association with HD (Supplementary Table S2). However, important genetic variants previously detected in studies associated with Alzheimer’s Disease were associated with HD (Supplementary Table S2, *p*-values < 0.05). Results shown in Supplementary Table S2, although associated with HD (those with *p*-values < 0.05) revealed that few sortilins SNP variants were in Linkage with HD (Supplementary Material Figure S3). Therefore, we investigated Linkage patterns unique to our GATK/GWAS dataset using the 665 SNPs shown in Figure 3 as associated with HD. A complete list of SNP variants per gene and identity of the 665 significant SNPs in *HTT* and sortilins genes are shown in Supplementary Materials Tables S8 and S9. After manually checking for the correlation of SNP position inheritance in the control and cases of HD, haplotype maps of the Linkage Disequilibrium analysis involving *HTT*, *SORT1*, *SORL1*, *SORCS1*, *2* and *3* SNPs variants are shown in Figure 5. 

Linkage patterns observed in the Labadorf dataset on Figure 5 were in agreement with the Linkage pattern observed in Lin 2016 (Supplementary Materials Figure S4). In both datasets, a continuous haplotype block including SNP Chr1:109950858 (indicated in the red square in Figure 5), upstream of *SORT1* is in association with another haplotype block in region Chr4:3236883-3238643 near *HTT* in HD cases, but not in controls of both Labadorf and Lin datasets (Figure 5, Supplementary Materials Figure S4). Genes, positions and *p*-values of SNPs associated with HD shown in Figure 5 are indicated in Supplementary Materials Table S10.

## 4. Discussion

Previously, we reported on the modulation in the expression of glucose transporters GLUT1 and GLUT4, and sortilin SORCS1 by mHTT in the ST14A cells, suggesting that mechanisms of insulin release and insulin sensitivity were affected in human HD by means of sortilin genes [19]. The identification of these genes in the HD pathology may be useful for therapeutic interventions that may include the CRISPR/Cas9 gene editing or small drug inhibition of such genes. Sortilin SORCS1 was described as a potential target in obesity. Sortilins are responsible for protein-protein interaction, internalization and sorting of proteins between the trans-Golgi network and endosomes using the retromer complex [14]. Given this role in cells, we hypothesized that sortilin plays a role in the release of exosome vesicles. To evaluate biological mechanisms of *SORCS1*, we are currently building a knock-out model of the *SORCS1* ST14A cells using CRISPR/Cas9 gene editing. We found non-coding variants in introns 1 and 2 of sortilin genes, including *SORCS1*, associated with HD in the human and in rat (Figure 1). This observation suggests that variants could affect the interaction of sortilins genomic sequence with proteins involved in their expression (Figure 1, Figure 2, Figure 3 and Figure 5). These results are in agreement with findings of Reitz et al., because these authors report on genetic variants located in introns 1 and 2 of *SORCS1*, associated with Alzheimer’s Disease [15]. We also found a total of 11 SNPs, three of which are significantly associated with HD, in common with SNPs observed by Reitz et al. 2013. All SNPs were found in the non-coding regions of sortilins, (significant SNPs, *p*-value < 0.05: *SORL1*, Chr11:121439665; *SORCS1*, Chr10:108706022 and *SORCS2*, Chr4:7733843) (Figure 1). *SORCS1* mutations located in intron 2, between exons that encode the VPS10P sorting domain (Figure 1). These SNPs are therefore common to HD and AD, suggesting that sortilins play biological roles in both AD and HD (Figure 1). We are confident about these results because the number of significant SNPs associated with HD was small compared to the total number of SNPs identified by the present GATK/GWAS pipeline (less than 5% in all datasets as shown in Table 1). Furthermore, we confirmed that the mutations detected by this GATK/GWAS pipeline were present in the genomic DNA derived from the ST14A cells using the pyrosequencing assay, ruling out the possibility of mRNA variation (Figure 4A).

The Labadorf group also reported modulation of sortilin *SORCS*3 by mHTT in their Supplementary Materials [28]. We observed that protein levels of sortilin SORCS1 were up-regulated in the ST14A cells (Figure 2A), suggesting modification in the normal subcellular traffic between intracellular organelles and plasma-membrane, as expected from cellular roles of sortilins [14,17]. Vázquez reported on the involvement of sortilins in the maturation of phagosomes in the process of phagocytosis of *Mycobacterium tuberculosis* [17]. In order to display peptides derived from this parasite in the MHC/HLA class II complex, endosomal and lysosomal enzymes need to be delivered in sequence (sorted) into the phagosome which contains the parasite proteins that will ultimately generate the parasite antigens [17]. Adaptor proteins are also implicated in the receptor-ligand transport of sortilins via clathrin-coated vesicles to endosomes. Once cargo is released, induced by the low-pH environment [44], the retrograde transport of sortilin from endosome to trans-Golgi network depends on the sortilin interaction with the retromer complex [14,17]. Therefore, results shown in Figure 1, Figure 2, Figure 3 and Figure 5 suggest that mHTT affects the expression or associate with genetic variants of sortilins in ST14A cells and human individuals. This is significant as it establishes a direct connection between vesicle traffic towards phagosome maturation for innate and adaptive immune responses and variants of sortilins involved in the process (Figure 3, Figure 5 and Supplementary Table S3). A further investigation of sortilins, tetraspanins and HLA/MHC genes, may lead to the understanding of the involvement of HTT and mHTT in the release of cellular vesicles, including exosomes, and how this might affect immunological pathways in diseases such as HD, diabetes and cancer.

One study reported an association of celiac disease (CE), a condition thought to primarily involve MHC/HLA genes, with *SORCS1* variants [45]. It is not surprising to find vesicle trafficking associated with defects in the HLA/MHC loci from the perspective of sortilins affecting vesicle trafficking in HD brought by our study. Before identification of mHTT as the cause of HD, one hypothesis was that the HLA/MHC loci were potential contributors or the only cause of HD. Consistent with this hypothesis, attempts were performed to assess the involvement of the HLA/MHC locus in the etiology of the Huntington’s Disease [46,47]. Although some association was initially reported, it did not hold true after scrutiny of statistical correction [46]. We observed several of the HLA/MHC rat genes with their expression influenced by mHTT in ST14A cells (data not shown). We are currently investigating the involvement of MHC/HLA genes in our ST14A HD cell model.

Our results add to the growing body of knowledge of sortilins as proteins that affect processing of antigens in autoimmunity and other functions related to the immune system by providing evidence that gene expression or genetic diversity of sortilin and MHC/HLA genes are associated with mHTT in HD (Figure 1, Figure 2, Figure 3 and Figure 5; Supplementary Materials Table S3). These findings contribute to an improved understanding on the control of traffic of glucose receptors, insulin and Alzheimer’s Disease proteins in the trans-Golgi network, the endosome and the plasma-membrane in normal and HD conditions, using models of HD described in this report (Figure 1, Figure 2, Figure 3, Figure 4 and Figure 5). We showed that mHTT is associated with increased protein expression of sortilin SORCS1 in ST14A cells (Figure 2A), in agreement with our previous report for mRNA molecules of SORCS1 and that there exists allele association (also known as Linkage Disequilibrium) of regions near SORT1, SORL1, SORCS1, SORCS2 and SORCS3 with gene variants in the region of HTT gene specifically in human HD cases (Figure 5). Eleven sortilins SNPs previously detected by Reitz et al. (2013), who investigated the involvement of SORCS1 variants with AD, were detected in our study (Supplementary Table S2) [14,15,16]. It is important to contrast the number of individuals involved in many GWAS studies to the number of HD cases reported here (Reitz 2013 individuals: *n* = 11,840 cases and 10,931 controls; Labadorf dataset 2015: *n* = 20 cases and 49 controls). Due to the sample size, more genetic variability is associated with the design of the Reitz group, which helps explain the low number of positions identified in common between the Labadorf and Reitz studies. Some of the SNP variants associated with HD were reported in Alzheimer’s and cardiovascular diseases GWASs (Supplementary Table S2). 

## 5. Conclusions

We present evidence of the association between the mHTT and genes of critical biological processes in neurodegenerative diseases, diabetes and cancer in rat and human RNA-Seq datasets. The genetic association of VPS10P (sortilins) variants with *HTT* variants in HD cases has also been established in this work. Genetic variance of the HLA/MHC loci in human and rat samples deserve further investigation, given the association with the mHTT shown in human and rat datasets. We hypothesize the existence of an allele association between sortilins and HLA/MHC genes. We envision sortilins and HLA/MHC proteins as potential therapeutic targets in cancer, diabetes, and neurodegenerative diseases, by modulation of antigen processing and immune response mechanisms in these diseases. In addition, our results further validate the rat ST14A cells as a model of human HD.

## Figures and Tables

**Table 1:** Summary of SNP variants detected in rat and human through genome analysis toolkit/genome-wide association study (RNA-Seq GATK/GWAS) pipeline. DNA variants identified in the gene body and in regulatory vicinity regions are indicated.

Dataset	Total Number of SNPs Identified	Significant SNPs (*p* < 0.05)	Sortilin Gene Body Size (bp)		SNPs in Gene Body (*p* < 0.05)	SNPs in Vicinity (±10^6^ bp Away from Gene Body)
*Rattus norvegicus (ST14A cells)*	222997	8464 (3.80%)	SORCS1	499515	6	13
			SORCS2	371129	0	8
			SORCS3	641740	0	2
			SORT1	89539	0	11
			SORL1	170117	0	5
Labadorf	2658838	84349 (3.17%)	SORCS1	590871	2	8
			SORCS2	550289	15	171
			SORCS3	624134	4	53
			SORT1	88381	9	153
			SORL1	181490	4	38
Lin	3172675	44669 (1.4%)	SORCS1	590871	6	9
			SORCS2	550289	10	121
			SORCS3	624134	0	23
			SORT1	88381	11	111
			SORL1	181490	4	41
HD iPSC Consortium (iPSC Cells)	238013	8979 (3.38%)	SORCS1	590871	0	0
			SORCS2	550289	0	20
			SORCS3	624134	0	3
			SORT1	88381	0	22
			SORL1	181490	3	6

## Supplementary Materials

The following are available online at https://www.mdpi.com/2073-4409/8/9/962/s1. Figure S1: Alignment of SORCS1 protein sequence from human, rat and mouse; Figure S2: Protein expression of SORCS1 in ST14A rat cells; Figure S3: Literature revised SNPs Linkage profile in HD Cases of Labadorf dataset; Figure S4: Linkage Disequilibrium of sortilin variants associated with Huntington’s Disease gene huntingtin (HTT) in cases of Lin 2016 dataset; Table S1: Significant SNPs associated with HD in all three human datasets; Table S2: Literature revised SNPs detected in sortilins in the present study; Table S3: Sequencing reads detected in sortilin and tetraspanin genes modulated or not by mutant huntingtin in iPSC-HD Consortium dataset; Table S4: Sequencing reads detected in sortilin genes of wild-type (WT) and mutant (MT) ST14A cells; Table S5: Student’s *t*-test results of SORCS1 protein expression in ST14A cells; Table S6: Sequencing reads detected by our group using DESeq2 in the datasets published by Labadorf et al., 2015; Table S7: Sequencing reads detected by our group using DESeq2 in the datasets published by Lin et al., 2016; Table S8: Total number of SNPs detected in vicinity of important loci for Huntington’s disease; Table S9: Identity of 665 SNPs significantly associated with HD, located near HTT and Sortilin genes; Table S10: Positions and *p*-values of SNPs in Linkage or allele association in Labadorf dataset.
