# Supplementary material for: Mutant Huntingtin Affects Diabetes and Alzheimer’s Markers in Human and Cell Models of Huntington’s Disease
Source: Cells. 2019 Aug 23;8(9):962. doi: 10.3390/cells8090962 (PMC6769852; doi:10.3390/cells8090962)
Supplement: Supplementary file 1 [file cells-08-00962-s001.pdf]

Supplementary Material for "MUTANT HUNTINGTIN AFFECTS DIABETES AND ALZHEIMER'S MARKERS IN HUMAN AND CELL MODELS OF HUNTINGTON'S DISEASE"

1) Unix Commands: processing of files for RNA-Seq, GWAS and Haploview plotting

Several steps of processing the files generated in this study were required using Unix commands. In this section, we organize the Unix code that was used for this purpose. Before each code line is stated, a brief sentence explaining what the goal of the code is will be provided. The commands are stated in somehow the sequence used in our analysis.

A) Isolate variants between gene coordinates. This command relies on the fact that the chromosome number is present in the output table generated by PLINK.

```
grep 'chromosome' plink_output | awk '$3 > Start ' | awk '$3 < End ' | awk '$8 < p_value'
```

B) Isolate significant SNPs from target dataset based on a list of SNP IDs. This command uses a list containing the chromosomal coordinates of each SNP position and greps the SNP position in a target dataset. This command was used to quickly check if a highly important SNP associated with diabetes in specific literature, for instance, was also detected in the vcf file containing all SNP variants detected in any of the RNA-Seq ST14A, Labadorf, Lin or HD IPSC Consortium datasets.

```
for i in $(cat SNPs_IDs_List.txt); do grep $i Target_Dataset; done
```

C) Use awk to isolate fields in columns. This awk command was used when we needed to specify a field separator, such as a comma, to isolate certain features of a gene, such as the coordinates of a gene.

```
awk 'BEGIN { FS = "," } ; { print $2 }'
```

D) Zip files, to comply with requirements of bcftools

```
for x in $(cat files_list.txt); do bgzip $x; done
```

E) Index files, to comply with requirements of bcftools

```
for x in $(cat files_list.txt); do tabix $x".gz"; done
```

F) Change directory to folder containing zipped files and indexes for those files, to run bcftools

```
cd Folder_Containing_Files
```

G) Merge vcf files to combined vcf file, using bcftools. Option --missing-to-ref considers empty genotypes as reference genotypes. This option is only available in version 1.9 of bcftools

```
bcftools merge --missing-to-ref --force-samples
SRR1747143_filtered_snps_final.vcf.gz
SRR1747144_filtered_snps_final.vcf.gz
SRR1747145_filtered_snps_final.vcf.gz
SRR1747146_filtered_snps_final.vcf.gz
SRR1747147_filtered_snps_final.vcf.gz
SRR1747148_filtered_snps_final.vcf.gz
SRR1747149_filtered_snps_final.vcf.gz
SRR1747150_filtered_snps_final.vcf.gz
SRR1747151_filtered_snps_final.vcf.gz
SRR1747152_filtered_snps_final.vcf.gz
SRR1747153_filtered_snps_final.vcf.gz
SRR1747154_filtered_snps_final.vcf.gz
SRR1747155_filtered_snps_final.vcf.gz
SRR1747156_filtered_snps_final.vcf.gz
SRR1747157_filtered_snps_final.vcf.gz
SRR1747158_filtered_snps_final.vcf.gz
SRR1747159_filtered_snps_final.vcf.gz
SRR1747160_filtered_snps_final.vcf.gz
SRR1747161_filtered_snps_final.vcf.gz
SRR1747162_filtered_snps_final.vcf.gz
SRR1747163_filtered_snps_final.vcf.gz
SRR1747164_filtered_snps_final.vcf.gz
SRR1747165_filtered_snps_final.vcf.gz
SRR1747166_filtered_snps_final.vcf.gz
SRR1747167_filtered_snps_final.vcf.gz
SRR1747168_filtered_snps_final.vcf.gz
SRR1747169_filtered_snps_final.vcf.gz
SRR1747170_filtered_snps_final.vcf.gz
SRR1747171_filtered_snps_final.vcf.gz
SRR1747172_filtered_snps_final.vcf.gz
SRR1747173_filtered_snps_final.vcf.gz
```

```
SRR1747174_filtered_snps_final.vcf.gz
SRR1747175_filtered_snps_final.vcf.gz
SRR1747176_filtered_snps_final.vcf.gz
SRR1747177_filtered_snps_final.vcf.gz
SRR1747178_filtered_snps_final.vcf.gz
SRR1747179_filtered_snps_final.vcf.gz
SRR1747180_filtered_snps_final.vcf.gz
SRR1747181_filtered_snps_final.vcf.gz
SRR1747182_filtered_snps_final.vcf.gz
SRR1747183_filtered_snps_final.vcf.gz
SRR1747184_filtered_snps_final.vcf.gz
SRR1747185_filtered_snps_final.vcf.gz
SRR1747186_filtered_snps_final.vcf.gz
SRR1747187_filtered_snps_final.vcf.gz
SRR1747188_filtered_snps_final.vcf.gz
SRR1747189_filtered_snps_final.vcf.gz
SRR1747190_filtered_snps_final.vcf.gz
SRR1747191_filtered_snps_final.vcf.gz
SRR1747192_filtered_snps_final.vcf.gz
SRR1747193_filtered_snps_final.vcf.gz
SRR1747194_filtered_snps_final.vcf.gz
SRR1747195_filtered_snps_final.vcf.gz
SRR1747196_filtered_snps_final.vcf.gz
SRR1747197_filtered_snps_final.vcf.gz
SRR1747198_filtered_snps_final.vcf.gz
SRR1747199_filtered_snps_final.vcf.gz
SRR1747200_filtered_snps_final.vcf.gz
SRR1747201_filtered_snps_final.vcf.gz
SRR1747202_filtered_snps_final.vcf.gz
SRR1747203_filtered_snps_final.vcf.gz
SRR1747204_filtered_snps_final.vcf.gz
SRR1747205_filtered_snps_final.vcf.gz
SRR1747206_filtered_snps_final.vcf.gz
SRR1747207_filtered_snps_final.vcf.gz
SRR1747208_filtered_snps_final.vcf.gz
SRR1747209_filtered_snps_final.vcf.gz
SRR1747210_filtered_snps_final.vcf.gz
SRR1747211_filtered_snps_final.vcf.gz
> Labadorf_merged.vcf
```

H) Use vcftools to extract ped and map from merged vcf file for plink downstream analysis

```
vcftools --vcf Labadorf_merged.vcf --out
formatted_plink_output --plink
```

I) Use plink to run Fisher's exact test statistics on all SNPs contained in the ped and map files

```
plink --noweb --ped formatted_plink_output.ped --map
formatted_plink_output.map --fisher
```

J) Call plink to extract targeted SNPs to run statistics on. Notice the –-extract and –-recode options, which allow specific extraction of a list of SNPs in text file snp_IDs

```
plink --noweb --ped formatted_plink_output.ped --map
formatted_plink_output.map --extract snp_IDs --recode --out
out_filtered_ped_map
```

K) After new ped file is formed, modify that file by opening using excel, then create new ped file for Cases and Controls. Use awk to modify the map file by extracting SNP name and genomic coordinate or position

```
awk '{print $2, $4}' map_file.map >
info_file_for_Haploview.info
```

L) Extract Chromosome, SNP name, Position and P-value columns from plink output to plot Manhattan plot in R

```
awk '{print $1, $2, $3, $8}' plink_output.fisher >
Manhattanly_Input.txt
```

Table S1. Significant SNPs associated with human HD detected in all three human datasets evaluated. These SNPs are the same indicated in the first Venn diagram in Figure 2B. Forty-three variants were considered significant ($p<0.05$, Fisher's exact test comparison) in the three datasets and are here displayed for reference (five of the SNPs indicated in Figure 2B did not match an Ensembl gene ID). The biological processes affected are well correlated with those described for HD by us and others.

SNP Position	Gene Coordinates	Gene descriptor	Ensembl ID
chr1:65450957	65,437,908-65,468,159	RP11-182I10.3	ENSG00000226891
chr1:10472848	10,458,649-10,480,201	phosphogluconate dehydrogenase	ENSG00000142657
chr1:25660621	25,568,728-25,664,704	chromosome 1 open reading frame 63	ENSG00000117616
chr1:45335074	45,316,450-45,452,282	eukaryotic translation initiation factor 2B, subunit 3 gamma, 58kDa	ENSG00000070785
**chr1:52383083**	**52,373,628-52,456,436**	**RAB3B, member RAS oncogene family**	**ENSG00000169213**
**chr1:65450956**	**65,450,291-65,451,770**	**solute carrier family 2 (facilitated glucose transporter), member 3 pseudogene 2**	**ENSG00000226891**
chr11:117054856	117,049,449-117,068,160	SID1 transmembrane family, member 2	ENSG00000149577
chr11:124015710	123,986,069-124,018,428	von Willebrand factor A domain containing 5A	ENSG00000110002
chr11:18893333	18,892,536-18,895,009	RP11-1081L13.3	ENSG00000255511
**chr11:2337012**	**2,323,239-2,339,430**	**tetraspanin 32**	**ENSG00000064201**
**chr12:31107955**	**31,079,682-31,145,086**	**tetraspanin 11**	**ENSG00000110900**
chr12:45459182	45,444,684-45,496,890	RP11-478B9.1	ENSG00000257319
chr12:54972992	54,943,134-54,973,023	phosphodiesterase 1B, calmodulin-dependent	ENSG00000123360
chr13:113889474	113,862,552-113,919,399	cullin 4A	ENSG00000139842
chr14:105642639	105,639,276-105,647,640	nudix (nucleoside diphosphate linked moiety X)-type motif 14	ENSG00000183828
chr14:45335074	45,329,519-45,335,450	CTD-2373J19.1	ENSG00000259000
chr14:75305174	75,230,069-75,322,244	YLP motif containing 1	ENSG00000119596
chr15:45459182	45,444,862-45,459,724	CTD-2651B20.1	ENSG00000259539
chr15:65298775	65,294,845-65,321,977	mitochondrial methionyl-tRNA formyltransferase	ENSG00000103707
chr16:53320257	53,088,945-53,363,062	chromodomain helicase DNA binding protein 9	ENSG00000177200
chr17:18133353	18,128,901-18,148,189	lethal giant larvae homolog 1 (Drosophila)	ENSG00000131899
chr17:74671208	74,668,633-74,707,098	matrix-remodelling associated 7	ENSG00000182534
**chr19:10793749**	**10,764,937-10,803,093**	**interleukin enhancer binding factor 3, 90kDa**	**ENSG00000129351**
chr19:49703690	49,660,998-49,715,093	transient receptor potential cation channel, subfamily M, member 4	ENSG00000130529
chr2:133070921	133,066,882-133,076,309	zinc finger protein 806	ENSG00000018607
chr2:60962386	60,962,030-60,962,850 r	ATPase, Na+/K+ transporting, beta 3 pseudogene	ENSG00000271707
chr2:99185423	99,061,317-99,210,853	inositol polyphosphate-4-phosphatase, type I, 107kDa	ENSG00000040933

**chr20:61992547**	**61,975,420-62,009,753**	**cholinergic receptor, nicotinic, alpha 4 (neuronal)**	**ENSG00000101204**
chr22:19974344	19,957,419-20,004,331	armadillo repeat gene deleted in velocardiofacial syndrome	ENSG00000099889
chr3:73022804	72,937,224-73,047,289	glucoside xylosyltransferase 2	ENSG00000172986
chr6:151336651	151,186,685-151,423,023	methylenetetrahydrofolate dehydrogenase (NADP+ dependent) 1-like	ENSG00000120254
**chr6:90011769**	**89,966,927-90,025,018**	**gamma-aminobutyric acid (GABA) A receptor, rho 2**	**ENSG00000111886**
chr6:99771540	99,728,543-99,790,810	failed axon connections homolog (Drosophila)	ENSG00000146267
**chr7:42959220**	**42,956,460-42,971,822**	**proteasome (prosome, macropain) subunit, alpha type, 2**	**ENSG00000106588**
chr7:44837298	44,836,279-44,864,163	peptidylprolyl isomerase A (cyclophilin A)	ENSG00000196262
chr8:63776566	63,161,150-63,912,211	Na+/K+ transporting ATPase interacting 3	ENSG00000185942
chr9:125450160	125,448,212-125,450,651	tousled-like kinase 1 pseudogene 1	ENSG00000226783
chr9:125450168	125,448,212-125,450,651	tousled-like kinase 1 pseudogene 1	ENSG00000226783
chr9:125450169	125,448,212-125,450,651	tousled-like kinase 1 pseudogene 1	ENSG00000226783
chr9:131222541	131,217,465-131,233,625	outer dense fiber of sperm tails 2	ENSG00000136811
**chr9:139702908**	**139,702,374-139,735,639**	**RAB, member RAS oncogene family-like 6**	**ENSG00000196642**

Table S2. Literature revised SNPs detected in sortilins in the present study. P-values of the association with HD are indicated, as well as study author of the reference text from which the SNP ID was collected.

dbSNP ID	Chr	Position	P-value in this study	Reference
**rs4970833**	**1**	**109804646**	**0.09685**	**Musunuru 2010**
rs653635	1	109806313	0.778	Musunuru 2010
rs6689614	1	109807099	0.1784	Musunuru 2010
rs2281894	1	109810544	0.647	Musunuru 2010
rs17035630	1	109810981	0.1795	Musunuru 2010
rs17035665	1	109813719	1	Musunuru 2010
rs4970834	1	109814880	1	Muendlein 2009, Musunuru 2010
rs611917	1	109815252	0.3212	Carrasquillo 2010, Musunuru 2010
**rs12740374**	1	109817590	0.8228	Musunuru 2010
rs660240	1	109817838	0.6518	Musunuru 2010
rs658435	1	109818270	1	Musunuru 2010
rs629301	1	109818306	0.8228	Musunuru 2010
**rs646776**	**1**	**109818530**	**0.00171**	**Muendlein 2009, Carrasquillo 2010, Musunuru 2010**
rs602633	1	109821511	0.7309	Musunuru 2010
**rs599839**	**1**	**109822166**	**0.1333**	**Muendlein 2009, Musunuru 2010, Linsel-Nitschke, 2010**
rs10410	1	109822404	1	Musunuru 2010
rs14000	1	109822509	1	Musunuru 2010
rs657420	1	109826136	1	Musunuru 2012
**rs672569**	**1**	**109827253**	**0.08497**	**Andersson 2016**
rs464218	1	109856306	1	Andersson 2016
rs17585355	1	109857815	1	Andersson 2016
rs7536292	1	109894693	1	Andersson 2016

rs11142	1	109897103	0.2947	Andersson 2016
rs72646553	1	109940503	0.5552	Carrasquillo 2010
rs17586966	1	109955569	1	Carrasquillo 2010, Reitz 2013
**rs12233824**	**4**	**7733843**	**0.005888**	**Reitz 2013**
rs3750261	10	107023390	0.8228	Reitz January 2011
rs7082289	10	108367020	1	Reitz 2013
rs10786997	10	108704547	0.232	Reitz 2013
rs11193128	10	108706198	0.3199	Reitz 2013
rs2149197	10	108716784	0.3199	Reitz January 2011
rs4918274	10	108729960	1	Rogaeva 2007, Lee 2007, Laumet 2010, Bettens 2007
rs578506	11	121323477	1	Rogaeva 2007, Laumet 2010, Bettens 2007, Louwersheimer 2015
rs12364988	11	121367626	0.3312	Reitz 2015, Vardarajan 2015
rs2298813	11	121393684	0.3199	Reitz 2013
**rs7946599**	**11**	**121423640**	**0.08497**	**Rogaeva 2007**
SORL1-T833T	11	121425955	1	Reitz 2013
**rs1784919**	**11**	**121439665**	**0.009079**	**Rogaeva 2007, Laumet 2010, Bettens 2007, Casingal, Kimura 2009**
rs2070045	11	121448090	0.6733	Rogaeva 2007, Lee 2007, Laumet 2010, Shibata, Bettens 2007
rs1699102	11	121456962	1	Reitz 2013
rs3824968	11	121475922	0.3339	Rogaeva 2007, Laumet 2010, Shibata, Bettens 2007, Kimura
rs2282649	11	121478958	0.5808	Rogaeva 2007, Laumet 2010, Shibata, Bettens 2007, Kimura
rs1010159	11	121483401	0.5441	Rogaeva 2007, Laumet 2010, Reitz 2013, Bettens 2007
rs1784933	11	121489416	0.5808	Rogaeva 2007, Laumet 2010, Bettens 2007, Louwersheimer 2015
rs1133174	11	121501755	0.5602	Rogaeva 2007, Laumet 2010, Bettens 2007, Louwersheimer 2015
rs1131497	11	121502745	0.7002	Musunuru 2010

2) Alignment of SORCS1 from human, rat and mouse

SORCS1 is a 130kDa protein highly conserved in human, rat and mouse. Sequence conservation can be seen in the protein alignment of sequences from the three species, using Clustal Omega.

```
CLUSTAL O(1.2.4) multiple sequence alignment

sp|Q8WY21|SORC1_HUMAN      MGKVGAGGGSQARLSALLAGAGLLILCAPGVCGGGSCCPSPHPSSAPRSASTPRGFSHQG 60
tr|F1LUZ4|F1LUZ4_RAT       MGKVGAGDGSSAALSALLTGAGLLMLLAPGICSSLSCCPPQHPSSTPRWTLTPRGFSYPG 60
sp|Q9JLC4|SORC1_MOUSE      MGKVGAGDGSSAGLSALLAGAGLLMLLAPGVCSSLSCCPPQHPSSTPRRTLTPRGFPHPG 60
                           *******.**.* *****:*****:* ***:*.. ****  ****:** : ***** : *

sp|Q8WY21|SORC1_HUMAN      RPGRAPATPLPLVVRPLFSVAPGDRALSLERARGTGASMAVAARSGRRRRSGADQEKAER 120
tr|F1LUZ4|F1LUZ4_RAT       PLGRAPATPPPLFMRPLFAVAPGDRALFLERAGGSRVSVATASRSGRRRRSGMDPEKTEP 120
sp|Q9JLC4|SORC1_MOUSE      PLGRAPATPPPLFMRPLFAVAPGDRALFLERAGGSRVSVATAARSGRRRRSGTEPEKIEP 120
                             ******* **.:****:******** **** *:  .*:*.*:********* : ** *

sp|Q8WY21|SORC1_HUMAN      GEGASRSPRGVLRDGGQQEPGTRERDPDKATRFRMEELRLTSTTFALTGDSAHNQAMVHW 180
tr|F1LUZ4|F1LUZ4_RAT       GEGTSRSRRDMLRDGGQQGPGTGARDPDKATRFRMEELRLTSTTFALTGDSAHNQAMVHW 180
sp|Q9JLC4|SORC1_MOUSE      GEGASRSRRDMLKDGGQQGLGTGARDPGKATRFRMEELRLTSTTFALTGDSAHNQAMVHW 180
                           ***:*** *.:*:*****  **  ***.********************************

sp|Q8WY21|SORC1_HUMAN      SGHNSSVILILTKLYDYNLGSITESSLWRSTDYGTTYEKLNDKVGLKTILSYLYVCPTNK 240
tr|F1LUZ4|F1LUZ4_RAT       SGHNSSVILILTKLYDYNLGSITESSLWRSTDYGTTYEKLNDKVGLKTILSYLYVCPTNK 240
sp|Q9JLC4|SORC1_MOUSE      SGHNSSVILILTKLYDYNLGSITESSLWRSTDYGTTYEKLNDKVGLKTILSYLYVCPTNK 240
                           ************************************************************

sp|Q8WY21|SORC1_HUMAN      RKIMLLTDPEIESSLLISSDEGATYQKYRLNFYIQSLLFHPKQEDWILAYSQDQKLYSSA 300
tr|F1LUZ4|F1LUZ4_RAT       RKIMLLTDPEIESSLLISSDEGATYQKYRLNFYIQSLLFHPKQEDWILAYSQDQKLYSSA 300
sp|Q9JLC4|SORC1_MOUSE      CKIMLLTDPEIESSLLISSDEGATYQKYRLNFYLQSLLFHPKQEDWILAYSQDQKLYSSA 300
                            ********************************:**************************

sp|Q8WY21|SORC1_HUMAN      EFGRRWQLIQEGVVPNRFYWSVMGSNKEPDLVHLEARTVDGHSYLTCRMQNCTEANRNQ 360
tr|F1LUZ4|F1LUZ4_RAT       EFGRRWQLIQEAVVPNRFYWSVLGSNKEPDLVHLEARTVDGHSIYLTCRMQNCTEANRNK 360
sp|Q9JLC4|SORC1_MOUSE      EFGRRWQLIQESVVPNRFYWSVMGSSKEPDLVHLEARTVDGHSIYLTCRMQNCTEANRNK 360
                           ***********.**********:**.***************** ***************:

sp|Q8WY21|SORC1_HUMAN      PFPGYIDPDSLIVQDHYVFVQLTSGGRPHYYVSYRRNAFAQMKLPKYALPKDMHVISTDE 420
tr|F1LUZ4|F1LUZ4_RAT       PFPGYIDPDSLIVQDDYVFVQLTSGGRPHYYVSYRRNPFAQMKLPKYALPKDMHVISTDE 420
sp|Q9JLC4|SORC1_MOUSE      PFPGYIDPDSLIVQDDYVFVQLTSGGRPHYYVSYRRSPFAQMKLPKYALPKDMHVISTDE 420
                           ***************.********************. **********************

sp|Q8WY21|SORC1_HUMAN      NQVFAAVQEWNQNDTYNLYISDTRGVYFTLALENVQSSRGPEGNIMIDLYEVAGIKGMFL 480
tr|F1LUZ4|F1LUZ4_RAT       NQVFAAVQEWNQNDTYNLYISDTRGVYFTLALENVQSSRGPEGNVMIDLYEVAGIKGMFL 480
sp|Q9JLC4|SORC1_MOUSE      NQVFAAVQEWNQNDTYNLYISDTRGVYFTLALENVRSSRGPEGNVMIDLYEVAGIKGMFL 480
                           ***********************************:********:***************

sp|Q8WY21|SORC1_HUMAN      ANKKIDNQVKTFITYNKGRDWRLLQAPDTDLRGDPVHCLLPYCSLHLHLKVSENPYTSGI 540
tr|F1LUZ4|F1LUZ4_RAT       ANKKIDNQVKTFITYNKGRDWRLLQAPDADLRGDPVHCLLPYCSLHLHLKVSENPYTSGI 540
sp|Q9JLC4|SORC1_MOUSE      ANKKIDNQVKTFITYNKGRDWRLLQAPDADLRGDPVHCLLPYCSLHLHLKVSENPYTSGI 540
                           ****************************:*******************************

sp|Q8WY21|SORC1_HUMAN      IASKDTAPSIIVASGNIGSELSDTDISMFVSSDAGNTWRQIFEEEHSVLYLDQGGVLVAM 600
tr|F1LUZ4|F1LUZ4_RAT       IASRDTAPSIIVASGNIGSELSDSDISMFVSSDAGNTWRQIFEEEHSILYLDQGGVLVAM 600
sp|Q9JLC4|SORC1_MOUSE      IASRDTAPSIIVASGNIGSELSDSDISMFVSSDAGNTWRQIFEEEHSILYLDQGGVLVAM 600
                           ***:*******************:***********************:************

sp|Q8WY21|SORC1_HUMAN      KHTSLPIRHLWLSFDEGRSWSKYSFTSIPLFVDGVLGEPGEETLIMTVFGHFSHRSEWQL 660
tr|F1LUZ4|F1LUZ4_RAT       KHTSLPIRHLWLSFDEGRSWSKYSFTSIPLFVDGVLGEPGEETLIMTVFGHFSHRSEWQL 660
sp|Q9JLC4|SORC1_MOUSE      KHTSLPIRHLWLSFDEGRSWSKYSFTSIPLFVDGVLGEPGEETLIMTVFGHFSHRSEWQL 660
                           ************************************************************
```


```
sp|Q8WY21|SORC1_HUMAN      KHTSLPIRHLWLSFDEGRSWSKYSFTSIPLFVDGVLGEPGEETLIMTVFGHFSHRSEWQL 660
tr|F1LUZ4|F1LUZ4_RAT       KHTSLPIRHLWLSFDEGRSWSKYSFTSIPLFVDGVLGEPGEETLIMTVFGHFSHRSEWQL 660
sp|Q9JLC4|SORC1_MOUSE      KHTSLPIRHLWLSFDEGRSWSKYSFTSIPLFVDGVLGEPGEETLIMTVFGHFSHRSEWQL 660
                           ************************************************************
```


```
sp|Q8WY21|SORC1_HUMAN      VKVDYKSIFDRRCAEEDYRPWQLHSQGEACIMGAKRIYKKRKSERKCMQGKYAGAMESEP 720
tr|F1LUZ4|F1LUZ4_RAT       VKVDYKSIFDRRCAEEDYRPWQLHSQGEACIMGAKRIYKKRKSERKCMQGTYAGAMESEP 720
sp|Q9JLC4|SORC1_MOUSE      VKVDYKSIFDRRCAEEDYRPWQLHSQGEACIMGAKRIYKKRKSERKCMQ-KYAGAMESEP 719
                           ************************************************* .*********

sp|Q8WY21|SORC1_HUMAN      CVCTEADFDCDYGYERHSNGQCLPAFWFNPSSLSKDCSLGQSYLNSTGYRKVVSNNCTDG 780
tr|F1LUZ4|F1LUZ4_RAT       CVCTEADFDCDYGYERHSSGQCLPAFWFNPSSLSKDCSLGQSYLNSTGYRKVVSNNCTDG 780
sp|Q9JLC4|SORC1_MOUSE      CVCTEADFDCDYGYERHSNGQCLPAFWFNPSSLSKDCSLGQSYLNSAGYRKVVSNNCTDG 779
                           ******************.***************************:*************

sp|Q8WY21|SORC1_HUMAN      VREQYTAKPQKCPGKAPRGLRIVTADGKLTAEQGHNVTLMVQLEEGDVQRTLIQVDFGDG 840
tr|F1LUZ4|F1LUZ4_RAT       VREQYTAKPQKCPGKAPRGLRIVTADGKLTAEQGHNVTLMVQLEEGDVQRTLIQVDFGDG 840
sp|Q9JLC4|SORC1_MOUSE      VREQYTAKPQKCPGKAPRGLRIVTADGKLTAEQGHNVTLMVQLEEGDVQRTLIQVDFGDG 839
                           ************************************************************

sp|Q8WY21|SORC1_HUMAN      IAVSYVNLSSMEDGIKHVYQNVGIFRVTVQVDNSLGSDSAVLYLHVTCPLEHVHLSLPFV 900
tr|F1LUZ4|F1LUZ4_RAT       IAVSYVNLSSMEDGIKHVYQNVGIFRVTVQVDNSLGSDSAVLYLHVTCPLEHVHLSLPFV 900
sp|Q9JLC4|SORC1_MOUSE      IAVSYVNLSSMEDGIKHVYQNVGIFRVTVQVDNSLGSDSAVLYLHVTCPLEHVHLSLPFV 899
                           ************************************************************

sp|Q8WY21|SORC1_HUMAN      TTKNKEVNATAVLWPSQVGTLTYVWWYGNNTEPLITLEGSISFRFTSEGMNTITVQVSAG 960
tr|F1LUZ4|F1LUZ4_RAT       TTKNKEVNATAVLWPSQVGTLTYVWWYGNNTEPLITLEGSISFKFTSEGMNTITVQVSAG 960
sp|Q9JLC4|SORC1_MOUSE      TTKNKEVNATAVLWPSQVGTLTYVWWYGNNTEPLITLEGSISFKFTSEGMNTITVQVSAG 959
                           *******************************************:****************

sp|Q8WY21|SORC1_HUMAN      NAILQDTKTIAVYEEFRSLRLSFSPNLDDYNPDIPEWRRDIGRVIKKSLVEATGVPGQHI 1020
tr|F1LUZ4|F1LUZ4_RAT       NAILQDTKTIAVYEEFRSLRLAFSPNLDDYNPDIPEWRRDISRVIKKSLVEATGIPSQHI 1020
sp|Q9JLC4|SORC1_MOUSE      NAILQDTKTIAVYEEFRSLRLAFSPNLDDYNPDIPEWRRDISRVIKKSLVEATGIPSQHI 1019
                           *********************:*******************.************:*.***

sp|Q8WY21|SORC1_HUMAN      LVAVLPGLPTTAELFVLPYQDPAGENKRSTDDLEQISELLIHTLNQNSVHFELKPGVRVL 1080
tr|F1LUZ4|F1LUZ4_RAT       LVAVLPGLPTAAELFVLPYQDGARENKRSPEDLEQISEVLIHKLNQNLVHFELKPGVQVL 1080
sp|Q9JLC4|SORC1_MOUSE      LVAVLPGLPTAAELFVLPYQDGTRENKRSPEDLEQISEVLIHKLNQNLVHFELKPGVQVL 1079
                           **********:********** : ***** :*******:***.**** *********:**

sp|Q8WY21|SORC1_HUMAN      VHAAHLTAAPLVDLTPTHSGSAMLMLLSVVFVGLAVFVIYKFKRRVALPSPPSPSTQPGD 1140
tr|F1LUZ4|F1LUZ4_RAT       VHAAHLTAAPLVDLTPTHSGSAMLMLLSVVFVGLAVFVIYKFKRRVALTSPPSPSTQPGD 1140
sp|Q9JLC4|SORC1_MOUSE      VHAAHLTAAPLVDLTPTHSGSAMLMLLSVVFVGLAVFVIYKFKRRVALPSPPSPSAQPGD 1139
                           ************************************************ ******:****

sp|Q8WY21|SORC1_HUMAN      SSLRLQRARHATPPSTPKRGSAGAQYAI 1168
tr|F1LUZ4|F1LUZ4_RAT       SSLRLQRPRQATPPSSPKRGSAGAQFAI 1168
sp|Q9JLC4|SORC1_MOUSE      SSLRLQRPRPATPPSSPKRGSAGAQFAI 1167
                           ******* * *****:*********:**
```

Figure S1. Alignment of SORCS1 protein sequence from human, rat and mouse using Clustal Omega. Color-coding represents physical chemical properties of the amino-acids side-chains: Pink – Positivelly charged; Blue – negatively charged; Green – Pollar Uncharged; Red – Hydrophobic.

2) Calculation of statistics on Western Blot and read counts using R package DESEq2 pipeline (Related to Figure 2C)

In this section, we present further assessment of sortilin data values in the ST14A, Labadorf et al. (2015), Lin et al. (2016) and HD iPSC Consortium RNA-seq datasets analysed in our study. Supplementary Table 1 shows RNA-Seq reads flanking sortilins, tetraspanins and HLA/MHC genes whose expression were found statistically significant in the HD iPSC Consortium dataset by DESeq2 (only *SORL1* was found statistically significant, as indicated in heatmap of Figure 2C, but the other sortilins were included in the table for comparison). RNA-Seq read count analysis was performed using R package DESeq2 as described before [1] and in our work involving mHTT in ST14A cells transcriptome.

Table S3. Sequencing reads detected in sortilin and tetraspanin genes modulated or not by mutant huntingtin in iPSC-HD Consortium dataset, corresponding to values used for heatmap on Figure 2C of the main text. Reads correspond to values plotted in heatmap of Figure 2C in main paper and were detected by our group using DESeq2 software. Expression of SORL1 mRNA was determined to be statistically significant in cells expressing mHTT by DESeq2 software.

	Number of Reads							Average		
**Gene**	**CTL1**	**CTL2**	**CTL3**	**HD1**	**HD2**	**HD3**	**HD4**	**Controls**	**HD Cases**	**Fold change**
SORL1	1222	857	542	152	394	283	384	874	303	0,3
SORT1	2854	1493	1418	1136	2721	1342	1522	1862	1725	0,9
SORCS1	810	290	330	156	552	369	510	477	397	0,8
SORCS2	464	560	755	214	597	844	647	696	498	0,7
SORCS3	41	283	127	106	247	37	125	136	139	1,0
TSPAN13	1197	447	1042	189	486	323	347	895	336	0,4
CD164	694	921	1219	577	2028	3400	3375	945	2345	2,5
CD2	0	1	0	11	29	20	14	1	19	19
CD200	598	309	387	73	218	38	112	431	110	0,3
CD248	36	149	456	348	1364	1503	3482	214	1674	7,8
CD34	9	108	18	102	239	354	616	45	328	7,3
CD37	151	78	67	71	186	283	190	99	183	1,8
CD59	728	667	949	410	1466	2511	2073	781	1615	2,1

CD63	1627	1983	1603	1069	4821	6189	6292	1738	4593	2,6
CD83	72	104	193	17	57	74	148	123	74	0,6
CD97	12	25	20	38	57	501	107	19	176	9,3
HLA-AS1	1	3	2	5	10	15	25	2	14	6,9
HLA-B	102	361	2	0	4	1	5	155	3	0,0

To contrast the data derived from the HD iPSC Consortium dataset with our ST14A rat model, Supplementary Table 2 shows number of reads found compatible with each sortilin gene. Note that only *SORCS1* was found statistically significant, as shown in previous work [2], and that *SORCS1* is up-regulated in ST14A cells. It is also important to note the small trend in *SORL1* values toward down-regulation, which was not significant (p-value<0.05).

Table S4. Sequencing reads detected in sortilin genes of wild-type (WT) and mutant (MT) cells derived from *Rattus norvegicus* (ST14A cells) and expressing mHTT, detected by our group using DESeq2 software.

	Number of Reads								Average		
**Gene**	**WT1**	**WT2**	**WT3**	**WT4**	**MT1**	**MT2**	**MT3**	**MT4**	**Controls (WT)**	**Mutant**	**Fold change**
SORL1_rat	1200	751	412	779	921	461	549	743	786	669	0,9
SORT1_rat	6	2	5	11	24	15	1	49	6	22	3,7
SORCS1_rat	0	0	0	0	391	306	226	252	1	294	293,8
SORCS2_rat	7	0	2	1	0	4	0	5	3	2	0,9
SORCS3_rat	2	0	0	0	0	0	0	1	1	0	0,5

3) SORCS1 Western Blot Statistics (Related to Figure 2A)

To compare *SORCS1* protein expression in wild-type and mutant ST14A cells, we calculated the band density of the higher molecular weight band on gel shown in Figure 2A of the main manuscript (~130kDa), and normalized the values using the intensity of tubulin bands (n = 3). Quantification of western blot bands was done using software Image J.

Supplementary Figure 2 shows quantification and normalization of the SORCS1 protein band, confirming up-regulation of SORCS1 gene, as we had demonstrated by qPCR and RNA-seq data.

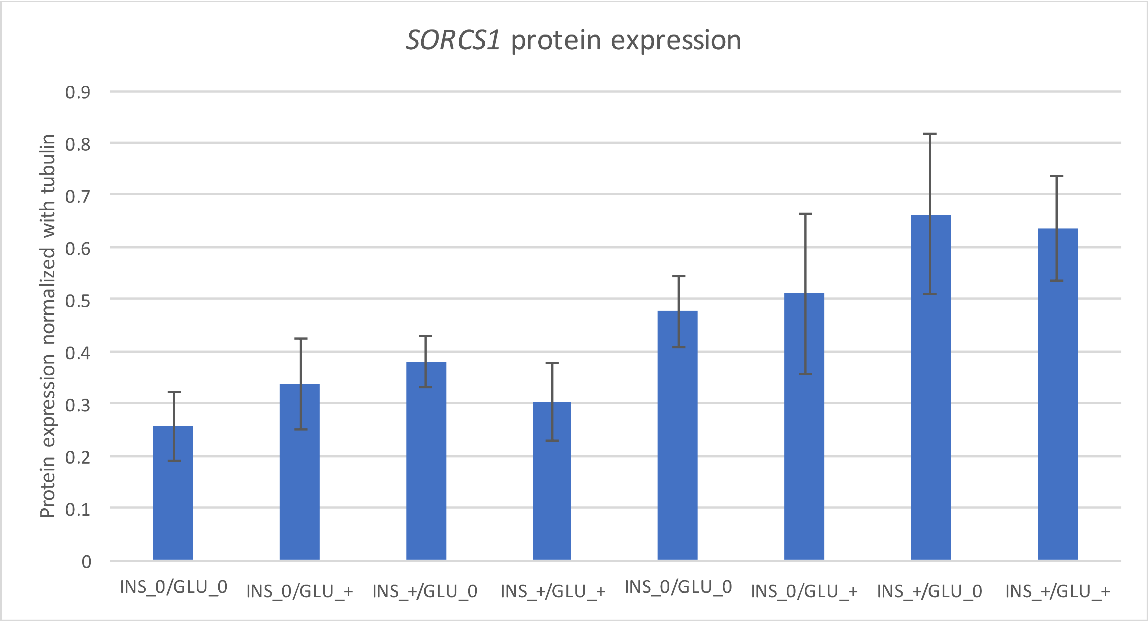

Figure S2. Protein expression of SORCS1 in ST14A rat cells receiving or not insulin and glucose treatments, as calculated using Image J for band quantification.

Supplementary Table 3 shows the student's t-test statistic calculated using the GraphPad Prism website (https://www.graphpad.com/quickcalcs/ttest2/).

Table S5. Student's t-test results of SORCS1 protein expression in ST14A cells (same replicates as Supplementary Figure 2), comparing replicates of wild-type and mutant ST14A cells.

	Average		
Group	***ST14A wild-type (Control)***	***ST14A Mutant***	***Fold Change***
Mean	0.31946325	0.57160317	1.789
SD	0.07666805	0.13444818	NA
SEM	0.02213216	0.03881185	NA
N	12	12	NA

Table S6. Sequencing reads (average) detected by our group using DESeq2 software and pipeline in the datasets published by Labadorf et al., 2015

	Average		
	**Controls**	**Cases**	**Fold Change**
SORT1	7737	7321	0.946
SORL1	5021	4389	0.874
SORCS1	731	634	0.867
SORCS2	893	582	0.651
SORCS3	506	353	0.697

Table S7. Sequencing reads (average) detected by our group using DESeq2 software and pipeline in the datasets published by Lin et al., 2016.

	Average		
	**Controls**	**Cases**	**Fold Change**
SORL1	87	66	0.759
SORT1	110	103	0.936
SORCS1	27	25	0.926
SORCS2	15	15	1
SORCS3	9	9	1

4) Manhattan plots of SNPs detected in Labadorf et al., 2015 dataset (Related to Figure 3)

Chromosome, SNP IDs, base pair position and P-value columns were isolated from Plink output of Fisher's exact association test using commands similar to the command described by sub-item a in item 1, illustrated above. Then, using the R package Qqman, Manhattan plots were produced highlighting SNPs in the SORT1 (chromosome 1), SORCS2 and HTT (both found on chromosome 4), SORCS1 and SORCS3 (both on chromosome 10), SORL1 (chromosome 11) and APOE (chromosome 19).

Table S8. Total number of SNPs detected in vicinity of important loci for Huntington's disease (these SNPs were considered significant as reflected by a p-value < 0.05).

<table>
<tr><th>Chromosome</th><th>Gene</th><th>Total SNPs in vicinity</th><th>Significant SNPs in vicinity</th></tr>
<tr><td>1</td><td>Sort1</td><td>4009</td><td>158</td></tr>
<tr><td>4</td><td>HTT</td><td rowspan="2">11746</td><td rowspan="2">365</td></tr>
<tr><td>4</td><td>Sorcs2</td></tr>
<tr><td>10</td><td>Sorcs1</td><td rowspan="2">1976</td><td rowspan="2">55</td></tr>
<tr><td>10</td><td>Sorcs3</td></tr>
<tr><td>11</td><td>Sorl1</td><td>1210</td><td>42</td></tr>
<tr><td>19</td><td>Apoe</td><td>1863</td><td>222</td></tr>
<tr><td colspan="3">Total HTT and Sortilins SNPs</td><td>665</td></tr>
</table>

5) Linkage Disequilibrium of literature SNPs associated with HD (Related to Supplementary Table 2)

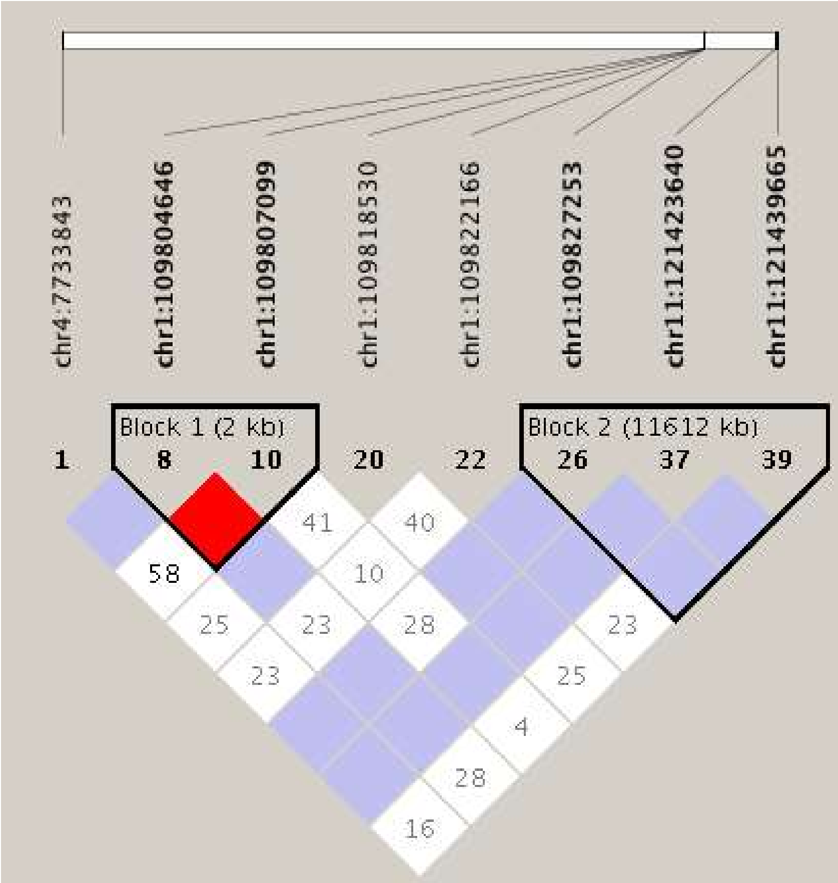

Figure S3. Literature revised SNPs Linkage profile in HD Cases of Labadorf dataset. Linkage blocks were found in the revised SNPs list, but the blocks involved only few SNP variants.

6) HD-associated SNPs in sortilin genes (Related to Figures 3 and 5 and Table 2)

Table S9. Identity of 665 SNPs significantly associated with HD, located near HTT and Sortilin genes. Includes SNPs indicated in Figures 3 and 5 and Supplementary Table 2.

CHR	Position	Minor allele name	Frequency of allele in cases	Frequency of allele in controls	Major allele name	Fisher's exact test p-value
1	109143318	A	0.075	0	T	0.0241
1	109170479	C	0	0.1042	T	0.03369
1	109192040	C	0.075	0	T	0.0241
1	109200205	T	0.1	0.3125	A	0.00908
1	109245303	G	0	0.1458	A	0.01031
1	109289487	T	0	0.1667	C	0.003152
1	109300742	T	0	0.1667	C	0.003152
1	109325549	G	0.2	0.07292	T	0.03938
1	109333576	C	0.15	0.02083	T	0.008231
1	109336411	A	0	0.1042	G	0.03369
1	109348095	G	0	0.1042	A	0.03369
1	109349647	G	0	0.1146	A	0.03336
1	109451676	G	0	0.1042	A	0.03369
1	109454285	C	0	0.1458	T	0.01031
1	109460068	G	0	0.1771	A	0.003059
1	109478125	G	0	0.1146	A	0.03336
1	109478265	G	0	0.125	A	0.0182

1	109482058	G	0	0.1354	A	0.01049
1	109495068	C	0	0.1042	T	0.03369
1	109495300	C	0	0.125	T	0.0182
1	109495310	C	0	0.1458	T	0.01031
1	109505689	C	0	0.125	T	0.0182
1	109514347	A	0.425	0.2396	C	0.03925
1	109515735	G	0	0.1042	A	0.03369
1	109515736	G	0	0.1042	T	0.03369
1	109524353	A	0.6	0.3333	G	0.006985
1	109554347	C	0.1	0	G	0.006703
1	109554349	C	0.1	0	A	0.006703
1	109605767	C	0.125	0.02083	T	0.02321
1	109606231	C	0.05	0.1979	T	0.03613
1	109606337	C	0.15	0.3333	T	0.03604
1	109606523	C	0.2	0.3854	T	0.04552
1	109635933	C	0.175	0.04167	G	0.01525
1	109710827	G	0	0.1042	C	0.03369
1	109734225	T	0.1	0	G	0.006703
1	109737224	C	0.15	0.02083	A	0.008231
1	109742485	A	0.175	0.4167	G	0.009533
1	109744815	G	0	0.1042	A	0.03369
1	109744828	G	0	0.1146	A	0.03336
1	109744835	G	0	0.1042	A	0.03369
1	109745292	G	0.125	0.03125	A	0.04818
1	109754223	G	0.1	0.01042	A	0.0262

1	109760312	G	0.025	0.1875	A	0.01305
1	109760324	G	0	0.1042	A	0.03369
1	109760641	G	0	0.1146	A	0.03336
1	109760668	G	0	0.125	A	0.0182
1	109761240	T	0.25	0.04167	C	0.0007465
1	109761255	A	0.25	0.05208	G	0.001746
1	109762236	G	0.175	0.4062	A	0.00996
1	109774026	G	0.55	0.1667	A	1.482e-05
1	109778237	T	0.05	0.2083	C	0.02225
1	109787851	G	0.1	0	A	0.006703
1	109791556	T	0.1	0	C	0.006703
1	109803892	G	0.075	0	T	0.0241
1	109803895	T	0.075	0	C	0.0241
1	109804252	G	0.2	0.0625	T	0.02708
1	109809110	A	0.325	0.5625	G	0.01446
1	109818530	T	0.325	0.09375	C	0.00171
1	109821307	T	0.2	0.04167	G	0.005888
1	109821588	A	0.1	0	G	0.006703
1	109836310	G	0.025	0.1562	C	0.03864
1	109836462	G	0	0.2396	A	0.0002326
1	109837357	G	0	0.1875	A	0.001613
1	109839433	A	0.6	0.375	G	0.02256
1	109841659	C	0.1	0.2812	T	0.02474
1	109841662	T	0.1	0.2812	A	0.02474
1	109860144	C	0.1	0.3021	T	0.01492

1	109868523	C	0	0.1042	T	0.03369
1	109868798	C	0	0.1771	T	0.003059
1	109870859	G	0.075	0.2292	C	0.04994
1	109878377	G	0	0.1042	C	0.03369
1	109891153	C	0	0.1146	T	0.03336
1	109891187	C	0	0.1458	T	0.01031
1	109891270	C	0.05	0.2188	T	0.02192
1	109915631	C	0.1	0	T	0.006703
1	109944078	T	0.6	0.3125	C	0.002221
1	109946169	C	0	0.1042	T	0.03369
1	109946176	C	0	0.1146	T	0.03336
1	109946774	C	0	0.1042	T	0.03369
1	109946882	C	0.175	0.4479	T	0.003168
1	109949008	G	0.2	0.3958	A	0.03014
1	109949746	C	0.025	0.1562	T	0.03864
1	109949748	C	0.025	0.1979	T	0.007605
1	109950607	G	0.15	0.4167	A	0.002758
1	109950858	C	0.05	0.2708	T	0.002553
1	109951711	C	0	0.2292	T	0.0002509
1	109952010	C	0	0.1042	T	0.03369
1	109952127	A	0.05	0.2188	G	0.02192
1	109952333	C	0	0.125	T	0.0182
1	110023432	A	0.15	0.02083	G	0.008231
1	110032751	T	0.275	0.1146	G	0.03818
1	110032753	C	0.3056	0.1341	A	0.03938

1	110032754	T	0.3	0.1146	A	0.0123
1	110040680	T	0.25	0.5	C	0.00799
1	110046489	G	0.075	0	A	0.0241
1	110121984	T	0.55	0.25	G	0.001292
1	110121992	C	0.2632	0.06977	T	0.006757
1	110121996	C	0.25	0.0625	T	0.006158
1	110128498	A	0.2	0.4062	T	0.02886
1	110163894	A	0.175	0.05208	T	0.04067
1	110163898	A	0.125	0.01042	T	0.008527
1	110169055	A	0.075	0	T	0.0241
1	110169056	G	0.125	0.02083	A	0.02321
1	110169059	T	0.075	0	G	0.0241
1	110169957	G	0.25	0.5	A	0.00799
1	110201809	T	0.25	0.4688	G	0.02153
1	110201812	G	0.25	0.4896	A	0.01286
1	110206242	G	0.1	0.01042	A	0.0262
1	110210780	T	0	0.1778	C	0.002733
1	110210782	C	0	0.1146	G	0.03336
1	110230532	C	0	0.1458	G	0.01031
1	110241057	G	0.15	0.02083	A	0.008231
1	110246036	C	0.3	0.1354	G	0.03025
1	110248468	G	0.2	0.07292	A	0.03938
1	110251746	A	0.425	0.1771	G	0.00421
1	110252010	T	0.075	0.3854	C	0.00017
1	110252091	A	0.35	0.1562	G	0.02021

1	110254971	C	0.05	0.3333	G	0.000362
1	110254977	T	0	0.1042	G	0.03369
1	110265499	C	0.1	0	T	0.006703
1	110275744	G	0.325	0.1562	A	0.03605
1	110282050	G	0	0.1146	C	0.03336
1	110318221	G	0.1	0	A	0.006703
1	110324411	C	0.075	0	T	0.0241
1	110469057	T	0.075	0	C	0.0241
1	110469411	G	0.175	0.04167	A	0.01525
1	110560566	T	0.2	0.0625	A	0.02708
1	110563814	A	0.425	0.2292	G	0.03625
1	110587924	A	0.3	0.0625	G	0.0004916
1	110587956	A	0.25	0.08333	G	0.01308
1	110589579	A	0	0.2292	G	0.0002509
1	110590816	T	0.1	0.3021	C	0.01492
1	110604747	A	0.475	0.2604	G	0.02597
1	110658039	C	0.1	0	T	0.006703
1	110674457	G	0.1	0	A	0.006703
1	110693190	G	0	0.1146	A	0.03336
1	110693263	T	0.05	0.2604	C	0.004263
1	110717590	G	0.1	0	C	0.006703
1	110720400	G	0.25	0.04167	A	0.0007465
1	110740406	T	0	0.1146	C	0.03336
1	110742502	G	0	0.1771	A	0.003059
1	110766534	A	0.025	0.1562	G	0.03864

1	110774447	G	0.15	0.3542	A	0.02241
1	110780501	A	0.1	0	C	0.006703
1	110790378	G	0.125	0.02083	A	0.02321
1	110799395	C	0	0.125	T	0.0182
1	110799403	C	0	0.125	T	0.0182
1	110799411	G	0	0.125	C	0.0182
1	110799412	G	0	0.125	A	0.0182
1	110799438	A	0	0.125	C	0.0182
1	110799443	G	0	0.125	T	0.0182
1	110909914	G	0.025	0.1771	A	0.0233
1	110910175	G	0	0.125	A	0.0182
1	110910187	G	0	0.1146	A	0.03336
1	110910211	G	0.1	0.2812	A	0.02474
1	110911787	G	0.025	0.1562	A	0.03864
1	110912071	G	0.075	0	A	0.0241
1	110916018	C	0.1	0	T	0.006703
4	2078853	C	0.05	0.2188	T	0.02192
4	2092075	C	0.025	0.2396	T	0.00232
4	2092138	T	0.125	0.3438	C	0.01133
4	2092145	G	0.125	0.3229	T	0.01902
4	2092151	G	0.1	0.3125	A	0.00908
4	2227633	C	0.075	0	T	0.0241
4	2252978	G	0.65	0.4375	T	0.03773
4	2252981	G	0.3	0.5521	C	0.008554

4	2252983	C	0.25	0.4479	A	0.03503
4	2269377	G	0.15	0.3542	C	0.02241
4	2271113	C	0.1	0.2604	G	0.04057
4	2276214	G	0	0.1562	T	0.00553
4	2277763	C	0	0.1042	T	0.03369
4	2306657	G	0.075	0	C	0.0241
4	2307121	A	0.025	0.1562	G	0.03864
4	2311719	C	0.025	0.1562	A	0.03864
4	2311863	C	0.075	0	T	0.0241
4	2341194	C	0	0.3125	T	7.154e-06
4	2344258	C	0	0.1146	T	0.03336
4	2344259	C	0	0.1146	T	0.03336
4	2371127	C	0	0.1667	T	0.003152
4	2371128	A	0	0.1667	G	0.003152
4	2371156	G	0	0.125	A	0.0182
4	2371174	C	0	0.1146	T	0.03336
4	2371930	G	0.1	0.2604	A	0.04057
4	2451694	C	0	0.1667	T	0.003152
4	2460571	T	0.05	0.1979	C	0.03613
4	2461888	G	0.3	0.5417	A	0.01389
4	2462508	C	0.05	0.2292	T	0.01284

4	2465304	A	0.05	0.25	G	0.007432
4	2492234	A	0	0.1146	G	0.03336
4	2652492	C	0.05	0.2292	T	0.01284
4	2655171	G	0	0.1146	A	0.03336
4	2656043	G	0	0.1042	A	0.03369
4	2656069	G	0	0.1042	A	0.03369
4	2657322	G	0	0.1042	A	0.03369
4	2665020	C	0.1053	0	G	0.005748
4	2717690	T	0.125	0.03125	C	0.04818
4	2733142	G	0.025	0.1667	A	0.02289
4	2749301	G	0.25	0.0625	C	0.006158
4	2826925	A	0.1	0	T	0.006703
4	2831883	C	0	0.125	G	0.0182
4	2831884	A	0	0.125	T	0.0182
4	2834047	A	0.075	0	T	0.0241
4	2839659	A	0.15	0.3333	G	0.03604
4	2841359	G	0	0.1042	A	0.03369
4	2911315	T	0	0.1042	C	0.03369
4	2923816	G	0	0.1771	T	0.003059
4	2935618	G	0.175	0.03125	C	0.007156
4	2941418	A	0.175	0.03125	G	0.007156

4	2944477	C	0.1	0.4792	T	1.469e-05
4	2944620	C	0	0.1354	T	0.01049
4	2960430	T	0.2	0.02083	C	0.0008888
4	2972604	A	0.325	0.5208	G	0.04018
4	2978620	C	0.1	0	T	0.006703
4	2986636	G	0	0.1146	A	0.03336
4	2987476	G	0.025	0.1562	A	0.03864
4	2989621	G	0.025	0.1771	A	0.0233
4	2989683	G	0	0.1354	A	0.01049
4	2991040	A	0	0.1146	G	0.03336
4	2991241	G	0.025	0.1875	A	0.01305
4	2998055	G	0.15	0	A	0.0004884
4	2998110	T	0.25	0.02083	C	8.07e-05
4	3023178	C	0.05	0.2083	T	0.02225
4	3033182	C	0	0.1667	T	0.003152
4	3033643	C	0.1	0	A	0.006703
4	3038415	G	0	0.25	A	0.0001192
4	3039241	G	0.075	0	A	0.0241
4	3039311	T	0.05	0.2812	C	0.002346
4	3041364	G	0	0.1458	A	0.01031
4	3043512	G	0	0.2292	C	0.0002509

4	3043513	C	0	0.2292	A	0.0002509
4	3048207	C	0.475	0.2292	T	0.007191
4	3224216	A	0.4	0.2188	T	0.03619
4	3231772	C	0.05	0.25	A	0.007432
4	3233844	G	0	0.1458	A	0.01031
4	3235081	C	0.125	0.02083	G	0.02321
4	3235084	C	0.125	0.02083	A	0.02321
4	3236881	G	0.05	0.25	T	0.007432
4	3236883	G	0.05	0.2292	A	0.01284
4	3241845	T	0.275	0.07292	C	0.003871
4	3243804	A	0.2	0.01042	G	0.0002288
4	3263138	A	0	0.1354	G	0.01049
4	3265130	C	0.05	0.2812	G	0.002346
4	3265710	T	0.075	0.2292	C	0.04994
4	3314646	G	0.1	0	A	0.006703
4	3377674	A	0	0.1042	G	0.03369
4	3380088	C	0.175	0.02083	T	0.002769
4	3409359	G	0	0.1146	A	0.03336
4	3411105	A	0	0.1042	G	0.03369
4	3411110	A	0	0.1562	G	0.00553
4	3415336	T	0.025	0.1667	C	0.02289

4	3415378	G	0	0.1354	A	0.01049
4	3438643	A	0.1	0	G	0.006703
4	3444593	A	0.125	0.03125	G	0.04818
4	3449886	A	0.475	0.125	G	2.684e-05
4	3473066	G	0	0.1146	A	0.03336
4	3476809	T	0.1	0	C	0.006703
4	3480439	T	0	0.125	C	0.0182
4	3485412	C	0	0.1042	T	0.03369
4	3487151	G	0.1	0.2604	A	0.04057
4	3496058	T	0.1	0.01042	C	0.0262
4	3496110	T	0	0.1354	C	0.01049
4	3506933	A	0.625	0.4271	G	0.04002
4	3508726	C	0.05	0.1979	T	0.03613
4	3508752	C	0.025	0.1979	T	0.007605
4	3510957	A	0.075	0.3229	G	0.002065
4	3512690	C	0.25	0.5208	T	0.004417
4	3517746	C	0.075	0	A	0.0241
4	3518190	T	0.2	0.0625	C	0.02708
4	3529671	A	0.1	0.01042	C	0.0262
4	3532327	C	0.15	0.03125	T	0.01915
4	3533066	T	0	0.1354	C	0.01049

4	3746133	C	0.15	0.02083	G	0.008231
4	3748134	A	0.075	0	C	0.0241
4	3765336	A	0.075	0.2396	G	0.03078
4	3944253	C	0.45	0.2396	T	0.02323
4	3944752	G	0.35	0.1562	A	0.02021
4	3944888	C	0.525	0.2917	T	0.01164
4	3946166	C	0.1	0.01042	T	0.0262
4	3946175	C	0.075	0	T	0.0241
4	3946290	C	0	0.1042	T	0.03369
4	3969218	T	0	0.1771	C	0.003059
4	4051294	G	0	0.1146	A	0.03336
4	4076788	C	0.15	0	T	0.0004884
4	4103099	C	0	0.1042	T	0.03369
4	4103104	C	0	0.2083	T	0.0008659
4	4103105	C	0	0.2083	T	0.0008659
4	4109198	C	0.25	0.4583	T	0.03372
4	4109210	C	0	0.1354	G	0.01049
4	4240627	G	0	0.1458	A	0.01031
4	4243668	C	0	0.1354	T	0.01049
4	4243684	T	0	0.1042	C	0.03369
4	4243862	C	0	0.1042	T	0.03369

4	4245210	C	0	0.1146	A	0.03336
4	4245510	C	0	0.1146	T	0.03336
4	4245513	C	0	0.1146	T	0.03336
4	4245591	T	0	0.1042	C	0.03369
4	4245926	A	0	0.1458	G	0.01031
4	4245929	A	0	0.1458	G	0.01031
4	4246109	C	0	0.1146	T	0.03336
4	4246433	C	0	0.1042	T	0.03369
4	4246453	C	0	0.125	T	0.0182
4	4246457	A	0	0.125	G	0.0182
4	4246497	C	0	0.125	T	0.0182
4	4249414	G	0.15	0.3542	A	0.02241
4	4249415	C	0.15	0.3542	T	0.02241
4	4249484	C	0.2	0.3958	A	0.03014
4	4271623	G	0.1	0.3542	C	0.002917
4	4275306	T	0.125	0.03125	A	0.04818
4	4304749	A	0.075	0	G	0.0241
4	4310432	G	0	0.1042	A	0.03369
4	4318931	A	0.15	0.3333	T	0.03604
4	4319564	C	0.15	0.3958	G	0.005044
4	4319728	C	0.05	0.375	A	4.849e-05

4	4319750	T	0.05	0.2083	C	0.02225
4	4322078	A	0.075	0.2708	G	0.01121
4	4422558	C	0	0.1042	A	0.03369
4	4553973	G	0.1	0.01042	A	0.0262
4	4641895	T	0	0.1042	G	0.03369
4	4732282	G	0.45	0.25	A	0.02608
4	4789635	A	0.075	0	G	0.0241
4	4824890	G	0	0.1771	A	0.003059
4	4825092	G	0	0.1146	A	0.03336
4	4865316	A	0.175	0.03125	G	0.007156
4	4865321	A	0.075	0	G	0.0241
4	5018702	A	0.1	0	G	0.006703
4	5812778	A	0.275	0.1146	G	0.03818
4	5814082	C	0.15	0.03125	G	0.01915
4	5815381	A	0.35	0.1875	G	0.04855
4	5833660	G	0	0.1354	C	0.01049
4	5833899	A	0	0.1562	T	0.00553
4	5839770	A	0	0.1042	G	0.03369
4	5843160	T	0.075	0	A	0.0252
4	5843163	G	0.075	0	C	0.0241
4	5843195	A	0.075	0	G	0.0241

4	5851205	T	0.15	0	G	0.0004884
4	5862752	C	0.5	0.2812	A	0.01809
4	5862938	T	0.4	0.1354	C	0.001115
4	5862943	C	0.1	0	G	0.006703
4	5901873	G	0.325	0.5312	A	0.03779
4	5905499	C	0.075	0.2292	T	0.04994
4	5906287	A	0.15	0.3438	G	0.02356
4	6018891	T	0	0.2604	C	6.608e-05
4	6019046	T	0	0.1667	C	0.003152
4	6020190	G	0.05	0.3542	A	0.0001057
4	6020367	T	0	0.1354	G	0.01049
4	6025638	T	0.15	0.3438	A	0.02356
4	6025656	C	0.125	0.3854	T	0.002347
4	6025766	G	0.2	0.4688	A	0.003743
4	6026058	A	0.175	0.3958	G	0.01594
4	6083488	C	0.1	0	G	0.006703
4	6204935	C	0.25	0.08333	T	0.01308
4	6238466	G	0.175	0.3646	A	0.04079
4	6239906	A	0.25	0.4583	G	0.03372
4	6240929	C	0.6	0.3854	T	0.0246
4	6245618	A	0.4	0.125	T	0.0007854

4	6245732	T	0.375	0.08333	G	9.731e-05
4	6245915	A	0.2	0.04167	T	0.005888
4	6246075	C	0.325	0.1458	G	0.03174
4	6246543	C	0.25	0.09375	T	0.02756
4	6246959	A	0.25	0.0625	G	0.006158
4	6270056	A	0	0.1042	G	0.03369
4	6290594	C	0.1	0.2604	T	0.04057
4	6292020	C	0	0.1562	A	0.00553
4	6294095	G	0	0.2188	A	0.0004494
4	6298375	T	0.025	0.1562	C	0.03864
4	6316092	T	0.075	0	C	0.0241
4	6321396	T	0.05	0.2188	C	0.02192
4	6324647	A	0.325	0.5312	G	0.03779
4	6324785	C	0.325	0.5521	T	0.02329
4	6327669	A	0	0.1354	C	0.01049
4	6328354	C	0	0.1042	G	0.03369
4	6328507	G	0	0.1354	A	0.01049
4	6333130	T	0.55	0.2708	C	0.002942
4	6333559	T	0.15	0.3333	G	0.03604
4	6333669	T	0.125	0.3229	G	0.01902
4	6335966	C	0	0.1042	T	0.03369

4	6435341	A	0	0.1667	G	0.003152
4	6435486	T	0.15	0.4375	C	0.001477
4	6437191	T	0	0.1667	A	0.003152
4	6437197	C	0	0.2292	T	0.0002509
4	6457121	C	0	0.125	A	0.0182
4	6457131	C	0	0.125	T	0.0182
4	6457132	A	0	0.125	G	0.0182
4	6568390	C	0.05	0.2083	A	0.02225
4	6570032	T	0.15	0.4375	C	0.001477
4	6570768	A	0.2	0.4062	G	0.02886
4	6596360	G	0.6	0.375	A	0.02256
4	6613252	G	0.225	0.0625	T	0.01286
4	6613462	G	0.1	0.01042	A	0.0262
4	6624771	G	0	0.125	A	0.0182
4	6626154	G	0	0.1042	A	0.03369
4	6641969	C	0	0.125	T	0.0182
4	6642090	T	0.15	0	C	0.0004884
4	6644466	A	0.125	0.03125	C	0.04818
4	6644467	A	0.125	0.03125	C	0.04818
4	6644468	A	0.175	0.04167	T	0.01525
4	6647889	G	0	0.1146	A	0.03336

4	6648300	T	0.375	0.1562	C	0.01132
4	6662665	C	0.1	0.01042	T	0.0262
4	6663715	C	0	0.1042	T	0.03369
4	6674554	C	0.325	0.1354	T	0.01593
4	6678553	A	0	0.125	G	0.0182
4	6678599	C	0	0.125	T	0.0182
4	6698664	A	0.125	0	G	0.001828
4	6698667	C	0.6	0.3333	T	0.006985
4	6698706	C	0.65	0.3125	A	0.0004859
4	6720572	T	0.1	0	A	0.006703
4	6860588	A	0.2	0.0625	G	0.02708
4	6874517	G	0	0.1042	A	0.03369
4	6911679	T	0	0.125	G	0.0182
4	6985889	C	0.55	0.1667	T	1.482e-05
4	6987394	T	0.45	0.2083	G	0.00622
4	7002344	G	0	0.125	A	0.0182
4	7004495	A	0	0.1562	G	0.00553
4	7004506	A	0	0.1562	G	0.00553
4	7005196	A	0	0.125	G	0.0182
4	7005199	G	0	0.125	C	0.0182
4	7024077	G	0	0.125	A	0.0182

4	7024398	G	0	0.1458	A	0.01031
4	7029430	G	0	0.1667	C	0.003152
4	7031064	C	0	0.1875	T	0.001613
4	7044357	A	0.75	0.3646	G	5.828e-05
4	7044380	T	0.1	0.4062	C	0.0004594
4	7048842	A	0.1	0	G	0.006703
4	7055253	T	0	0.125	C	0.0182
4	7064243	T	0.1	0	C	0.006703
4	7067765	G	0.175	0.02083	A	0.002769
4	7073187	G	0	0.1354	A	0.01049
4	7074027	G	0.25	0.08333	A	0.01308
4	7677967	A	0.1	0	G	0.006703
4	7701947	G	0	0.2188	A	0.0004494
4	7702795	A	0	0.1146	G	0.03336
4	7703505	C	0	0.2188	T	0.0004494
4	7703807	T	0.05	0.3229	C	0.0003864
4	7704795	T	0.025	0.2292	C	0.002389
4	7704818	T	0.025	0.2188	G	0.004156
4	7709703	A	0	0.1354	G	0.01049
4	7712150	C	0	0.125	T	0.0182
4	7712806	C	0	0.1042	T	0.03369

4	7714490	T	0.5	0.2917	C	0.02973
4	7733843	G	0.2	0.04167	A	0.005888
4	7735162	C	0.1	0	A	0.006703
4	7735164	G	0.1	0	C	0.006703
4	7736103	A	0.1	0	C	0.006703
4	7736112	A	0.1	0	T	0.006703
4	7744664	A	0.125	0.3438	G	0.01133
4	7753589	C	0	0.1042	T	0.03369
4	7774064	G	0	0.1042	C	0.03369
4	7783163	A	0.225	0.4167	G	0.04916
4	7820902	G	0	0.1042	T	0.03369
4	7820905	C	0	0.1042	A	0.03369
4	7876390	G	0	0.1042	A	0.03369
4	7876410	C	0	0.1042	T	0.03369
4	7959631	C	0	0.1354	T	0.01049
4	7959646	C	0	0.1354	T	0.01049
4	7959813	T	0.525	0.2708	C	0.005814
4	7959824	A	0.625	0.3438	G	0.004022
4	7961442	C	0	0.1667	T	0.003152
4	7961448	C	0	0.1146	T	0.03336
4	7967683	T	0.225	0.0625	C	0.01286

4	7967875	A	0.225	0.0625	G	0.01286
4	7968241	A	0.225	0.0625	G	0.01286
4	7969594	A	0.125	0.3021	G	0.03145
4	7970749	T	0.1	0.01042	C	0.0262
4	7974048	C	0	0.1146	T	0.03336
4	7977943	T	0	0.1042	C	0.03369
4	7978223	C	0	0.1042	T	0.03369
4	7990030	A	0.075	0	G	0.0241
4	8009759	A	0.175	0.4062	G	0.00996
4	8011632	G	0.05	0.1979	A	0.03613
4	8012665	G	0	0.1042	A	0.03369
4	8015869	G	0	0.1875	A	0.001613
4	8026166	G	0.05	0.3646	C	9.303e-05
4	8026796	C	0	0.1146	A	0.03336
4	8027827	C	0.1	0.2917	T	0.01576
4	8028963	T	0.1	0.2812	A	0.02474
4	8031471	A	0.4	0.2188	G	0.03619
4	8096147	A	0.125	0.2917	C	0.04813
4	8155197	T	0	0.1042	C	0.03369
4	8207676	A	0.1	0	G	0.006703
4	8207810	T	0.1	0	C	0.006703

4	8209447	A	0.1	0	C	0.006703
4	8220029	A	0.075	0	G	0.0241
4	8229326	T	0.2	0.5	C	0.001174
4	8230893	T	0.1	0	C	0.006703
4	8232966	G	0	0.1667	A	0.003152
4	8242499	A	0.1	0.01042	G	0.0262
4	8362394	C	0.075	0	T	0.0241
4	8362921	C	0	0.1146	T	0.03336
4	8363031	C	0.1	0.2604	T	0.04057
4	8363101	C	0.05	0.25	T	0.007432
4	8363109	C	0	0.1562	T	0.00553
4	8365580	C	0	0.1042	T	0.03369
4	8467291	A	0.6	0.3438	C	0.007567
4	8471398	C	0	0.1042	T	0.03369
4	8484413	T	0.05	0.2188	C	0.02192
4	8485146	C	0	0.1042	G	0.03369
4	8489819	C	0	0.1042	A	0.03369
4	8490793	A	0.075	0	C	0.0241
4	8493065	C	0.2	0.4062	G	0.02886
4	8495435	T	0.025	0.1667	C	0.02289
4	8496475	C	0.1	0	G	0.006703

4	8496689	T	0.15	0.02083	C	0.008231
4	8499118	G	0.075	0	A	0.0241
4	8505410	A	0	0.1667	T	0.003152
4	8511223	G	0.3	0.5104	A	0.03672
4	8512529	A	0.05	0.2917	G	0.001326
4	8517314	G	0.1	0	A	0.006703
4	8518431	G	0	0.1146	A	0.03336
4	8536551	G	0	0.1667	A	0.003152
4	8536913	T	0.15	0.03125	C	0.01915
4	8590967	T	0	0.1042	C	0.03369
4	8594574	C	0.3	0.02083	T	6.271e-06
4	8594577	T	0.125	0	C	0.001828
4	8595959	A	0.1	0	G	0.006703
4	8602816	G	0.25	0.0625	A	0.006158
4	8602884	C	0.35	0.1458	T	0.01047
4	8602935	G	0.35	0.1458	A	0.01047
4	8607587	A	0.05	0.25	G	0.007432
4	8607921	G	0.075	0	C	0.0241
4	8609153	A	0.1	0.01042	G	0.0262
4	8611409	C	0	0.1354	G	0.01049
4	8621194	G	0.55	0.3542	A	0.03791

10	105428963	T	0	0.1562	C	0.00553
10	105429020	T	0.1	0.4375	A	0.0001175
10	105432294	A	0.1	0.3021	G	0.01492
10	105615074	T	0	0.125	C	0.0182
10	105615105	C	0.05	0.3125	G	0.0006898
10	105624780	A	0	0.1042	G	0.03369
10	105627051	C	0	0.1458	T	0.01031
10	105630026	G	0.05	0.2083	T	0.02225
10	105677897	C	0	0.25	A	0.0001192
10	105777307	G	0	0.1354	A	0.01049
10	105777338	G	0	0.1042	A	0.03369
10	105779682	A	0	0.1146	T	0.03336
10	105783017	T	0	0.125	G	0.0182
10	105882845	C	0.1	0	G	0.006703
10	105995197	G	0	0.125	A	0.0182
10	105995200	G	0	0.125	T	0.0182
10	106014467	A	0.15	0.4271	G	0.00267
10	106022842	C	0.475	0.2708	A	0.02786
10	106022844	A	0.475	0.2708	T	0.02786
10	106027214	A	0.45	0.25	T	0.02608
10	106027216	A	0.4	0.1042	T	0.0001831
10	106027217	A	0.4	0.1042	G	0.0001831
10	106034491	G	0.1	0	A	0.006703
10	106035103	A	0.05	0.2083	G	0.02225
10	106035104	T	0.075	0	G	0.0241

10	106039817	G	0.075	0	A	0.0241
10	106039830	G	0.075	0	A	0.0241
10	106040086	G	0.1	0	A	0.006703
10	106040417	C	0	0.125	T	0.0182
10	106042237	G	0	0.1146	A	0.03336
10	106048969	G	0	0.1562	A	0.00553
10	106056152	G	0.05	0.25	A	0.007432
10	106056293	G	0.025	0.1562	A	0.03864
10	106056314	G	0.1	0.375	A	0.0009804
10	106056318	G	0.025	0.1875	A	0.01305
10	106056615	G	0.1	0.2917	A	0.01576
10	106056703	G	0	0.1458	A	0.01031
10	106056771	G	0	0.1771	A	0.003059
10	106056817	G	0.1	0.2604	A	0.04057
10	106058778	T	0.275	0.09375	C	0.01437
10	106061951	G	0.1	0.3646	A	0.001654
10	106063393	G	0.15	0.02083	A	0.008231
10	107016684	A	0.125	0.03125	T	0.04818
10	107016686	A	0.125	0.03125	T	0.04818
10	107016687	A	0.125	0.01042	G	0.008527
10	107021332	T	0.05	0.2396	C	0.007778
10	107437321	C	0.05	0.25	T	0.007432
10	107446263	A	0.15	0.3438	G	0.02356
10	107446373	A	0.425	0.2292	G	0.03625
10	107446398	T	0.225	0.04167	C	0.002151

11	120827609	A	0.525	0.2917	G	0.01164
11	120996292	G	0	0.1562	A	0.00553
11	121008734	C	0	0.1458	G	0.01031
11	121008736	A	0	0.1458	G	0.01031
11	121008737	C	0	0.125	T	0.0182
11	121032978	A	0.3	0.5729	G	0.004672
11	121038810	T	0	0.1042	C	0.03369
11	121233814	T	0.05	0.2083	C	0.02225
11	121411041	G	0.05	0.1979	A	0.03613
11	121416828	G	0	0.1042	T	0.03369
11	121439665	C	0.3	0.1042	T	0.009079
11	121478457	A	0.2	0.0625	G	0.02708
11	121490460	T	0.1	0.2708	A	0.03968
11	121491948	T	0.1	0.2708	G	0.03968
11	121799300	T	0.1	0	G	0.006703
11	121899825	C	0.3	0.125	T	0.02452
11	121902595	A	0.45	0.25	T	0.02608
11	121905237	G	0.2	0.5417	A	0.0002775
11	121908053	A	0	0.1042	G	0.03369
11	121911100	G	0.1	0.2917	A	0.01576
11	121913183	T	0.05	0.2292	C	0.01284
11	121913807	T	0.1	0.2708	C	0.03968
11	121917170	T	0	0.125	C	0.0182

11	121935860	C	0.25	0.5729	T	0.0006693
11	121938527	G	0	0.1146	T	0.03336
11	121968942	G	0.7	0.375	A	0.000665
11	122484733	G	0.1	0.4792	C	1.469e-05
11	122484878	C	0.25	0.04167	G	0.0007465
11	122484953	T	0.05	0.2708	C	0.002553
11	122484973	A	0.05	0.2708	T	0.002553
11	122484979	C	0.05	0.2708	T	0.002553
11	122485004	T	0.05	0.2708	C	0.002553
11	122485016	T	0.05	0.2708	C	0.002553
11	122489614	C	0.45	0.25	T	0.02608
11	122489631	A	0.65	0.3542	G	0.002263
11	122489638	T	0.65	0.375	C	0.004474
11	122489643	A	0.65	0.375	G	0.004474
11	122489691	G	0.65	0.375	A	0.004474
11	122489700	G	0.6	0.375	C	0.02256
11	122489709	A	0.4	0.2083	G	0.03192
11	122489714	C	0.35	0.1875	T	0.04855
11	122489929	T	0	0.125	C	0.0182

7) Linkage Disequilibrium of HD-associated SNPs

(SNPs showed to Figure 6)

Table S10. Positions and *p*-values of SNPs in Linkage or allele association in Labadorf dataset. These are the SNPs significantly associated with HD, indicated on Figure 5 of the main text.

Chr	Gene	Position	p-value Fisher's test
4	HTT	3048207	0.007191
4	HTT	3236883	0.01284
4	HTT	3241845	0.003871
4	HTT	3243804	0.0002288
4	HTT	3265130	0.002346
4	HTT	3265710	0.04994
4	HTT	3438643	0.006703
4	HTT	3510957	0.002065
4	SORCS2	7735162	0.006703
4	SORCS2	7735164	0.006703
4	SORCS2	7744664	0.01133
4	SORCS2	7783163	0.04916
4	SORCS2	7967683	0.01286
4	SORCS2	7967875	0.01286
4	SORCS2	7968241	0.01286
4	SORCS2	8011632	0.03613
4	SORCS2	8207676	0.006703
4	SORCS2	8207810	0.006703
4	SORCS2	8209447	0.006703
4	SORCS2	8490793	0.0241
10	SORCS1	107437321	0.007432
1	SORT1	109841659	0.02474
1	SORT1	109841662	0.02474

1	SORT1	109950858	0.002553
1	SORT1	109952127	0.02192
11	SORL1	121233814	0.02225
11	SORL1	121411041	0.03613
11	SORL1	122484953	0.002553
11	SORL1	122484973	0.002553
11	SORL1	122484979	0.002553

8) Linkage Disequilibrium of Labadorf HD-associated SNPs in Lin dataset (Related to Figure 6)

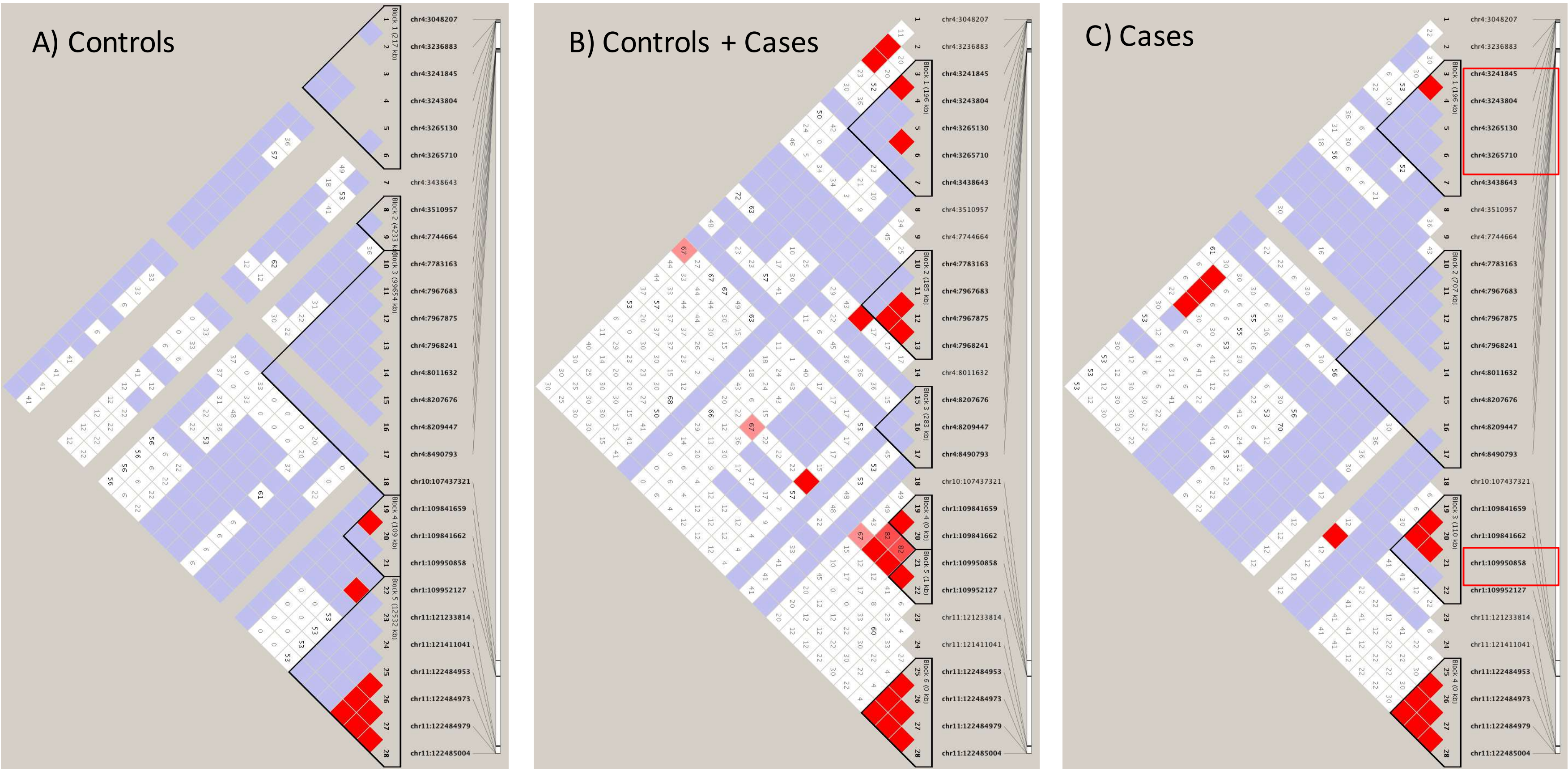

Figure S4. Linkage Disequilibrium or allele association of sortilin variants with Huntington's Disease gene huntingtin (HTT) in human cases of Lin 2016 dataset. Haplotype blocks indicated in red squares around chromosome positions of HTT and Sortilin genes present Linkage according to Haploview analysis in HD Cases, but not in control individuals. These results are in agreement with Figure 6 of the main text.

References

1. *Anders, S.; Huber, W. Differential expression analysis for sequence count data.* Genome Biol ***2010***, 11, *R106.*

2. *Chaves, G.; Özel, R.; Rao, N.V.; Hadiprodjo, H.; Costa, Y.; Tokuno, Z.; Pourmand, N. Metabolic and transcriptomic analysis of Huntington's disease model reveal changes in intracellular glucose levels and related genes.* Heliyon **2017**, 3, *e00381.*
